# Microbiota–gut–brain axis and bile acids–driven neuromodulation

**DOI:** 10.4103/NRR.NRR-D-25-00927

**Published:** 2025-12-30

**Authors:** Taiwei Dong, Tianyi Zhang, Huanhuan Wang, Jing Zhang, Reema Abdullah, Binggui Sun, Guoping Peng

**Affiliations:** 1 Department of Neurology, The First Affiliated Hospital, Zhejiang University School of Medicine, Hangzhou, Zhejiang Province, China; 2 School of Basic Medical Sciences, Hangzhou Normal University, Hangzhou, Zhejiang Province, China; 3 Department of Pathology, The First Affiliated Hospital, Zhejiang University School of Medicine, Hangzhou, Zhejiang Province, China; 4 Zhejiang University School of Medicine, Hangzhou, Zhejiang Province, China; 5 Department of Neurobiology and Department of Anesthesiology, the Children’s Hospital, Zhejiang University School of Medicine and National Clinical Research Center for Child Health; NHC and CAMS Key Laboratory of Medical Neurobiology, School of Brain Science and Brain Medicine, Zhejiang University, Hangzhou, Zhejiang Province, China

**Keywords:** Alzheimer’s disease, amyotrophic lateral sclerosis, bile acids, central nervous system, Huntington’s disease, microbiota-gut-brain axis, neurodegenerative diseases, Parkinson’s disease, sexual dimorphism, therapeutics

## Abstract

Bile acids emerge as multifunctional signaling molecules with dual hepatic and microbial origins, acting through farnesoid X receptor and Takeda G protein-coupled receptor 5 to influence inflammation and metabolism. Their dysregulation is consistently observed across various neurodegenerative diseases. The microbiota–gut–brain axis is a pivotal conduit for bile acids-driven neuromodulation, while sex-specific bile acid profiles and signaling pathways introduce critical biological heterogeneity. Emerging translational evidence indicates the promise of bile acids as biomarkers and therapeutic targets, yet highlights the critical hurdles that need to be addressed to realize precision interventions. Our core findings are: (1) Bile acids are far more than mere metabolic byproducts. They orchestrate core pathological processes such as neuroinflammation and energy metabolism. Their functions, whether neuroprotective or neurotoxic, are highly context-dependent, varying with cell type and disease-specific pathological backgrounds, thus exhibiting a potent “double-edged sword” effect. (2) The “microbiota–bile acids–brain axis” serves as a crucial bridge linking peripheral metabolic dysregulation to central nervous system pathology. (3) Sexual dimorphism emerges as a fundamental biological variable essential for understanding the heterogeneity in bile acid profiles and disease susceptibility. The primary contribution of this work is the proposal of an integrated “microbiota-bile acids-sex” framework that systematically describes the key scientific challenge of the context-dependent, dual roles of bile acids. Ultimately, this review champions a paradigm shift from a traditional brain-centric view to a systemic, metabolic perspective, establishing the bile acid system as a promising target for future precision therapeutic interventions.

**Facts**

• The gut microbiota is the core engine of bile acid biotransformation. It creates a complex and functionally diverse pool of secondary bile acids. These secondary bile acids act as key signaling molecules along the microbiota–gut–brain axis, influencing the central nervous system.

• Bile acid metabolism is highly consistent across various neurodegenerative diseases, but its effects depend on the specific pathological context. Bile acids have dual effects—both neuroprotective and neurotoxic—and exhibit disease-specific dysregulation patterns, highlighting the need for precision medicine approaches in therapeutic development.

• Sex is a key yet underestimated biological variable that influences the entire microbiota–bile acid–brain axis. Differences in bile acid profiles driven by factors such as hormones and the microbiota may explain the sex differences in incidence and progression of neurodegenerative diseases such as Alzheimer’s disease.

**Open questions**

• What is the composition and function of the endogenous bile acid pool in the brain? How do these brain-derived bile acids cooperate with peripheral bile acids to regulate neuronal function under different physiological conditions?

• Is bile acid dysregulation a driving factor of neurodegenerative diseases or a consequence?

• How can we harness the benefits of bile acid signaling in treatment while avoiding its toxic effects?

## Introduction

**Figure 1:**
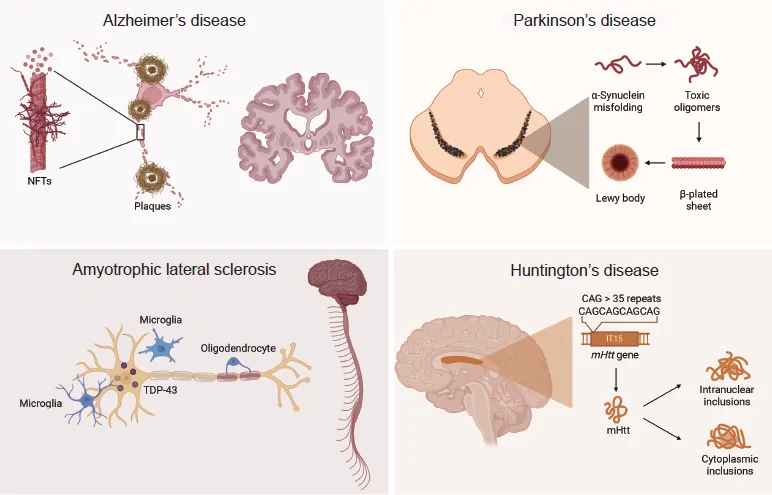
Key pathological features of major neurodegenerative diseases. This schematic illustrates the defining characteristics of Alzheimer’s disease, Parkinson’s disease, amyotrophic lateral sclerosis, and Huntington’s disease. Alzheimer’s disease shows extracellular plaques and intracellular NFTs. Parkinson’s disease is characterized by α-synuclein misfolding leading to toxic oligomers and Lewy body formation. Amyotrophic lateral sclerosis involves TDP-43 inclusions and glial cells such as microglia and oligodendrocytes. Huntington’s disease is caused by *mHtt* gene with CAG > 35 repeats, leading to mHtt protein aggregation and subsequent intranuclear and cytoplasmic inclusions. CAG: Cytosine-adenine-guanine; mHtt: mutant huntingtin protein; NFTs: neurofibrillary tangles; TDP-43: TAR DNA-binding protein 43.

Neurodegenerative diseases, including Alzheimer’s disease (AD), Parkinson’s disease (PD), amyotrophic lateral sclerosis (ALS), and Huntington’s disease (HD), are an escalating public health burden in an aging population (Cheng et al., 2025; Song et al., 2025). Despite their clinical heterogeneity, a convergent view is emerging: these disorders are anchored in shared molecular disruptions, notably protein misfolding, abnormal aggregation, and chronic neuroinflammation (**Figure 1**; Boskovic et al., 2024; Xu et al., 2025). This perspective is expanding beyond the traditional central nervous system (CNS)-centric framework, as researchers increasingly examine the fundamental processes of neural injury, repair, and regeneration from a systemic, whole-body perspective. A pivotal shift in the field now recognizes the microbiota-gut-brain axis (MGBA) as a critical regulatory network through which gut-derived signals can influence CNS homeostasis (Wang et al., 2023). Among these signals, bile acids (BAs)—once considered digestive aids—have emerged as versatile signaling molecules capable of modulating core neuropathological processes (Zhao et al., 2025).

Current research indicates that BAs, whose chemical diversity is expanded by the gut microbiota, affect neuroinflammation, mitochondrial function, and blood–brain barrier (BBB) integrity through receptors such as farnesoid X receptor (FXR) and Takeda G protein-coupled receptor 5 (TGR5). Several studies link abnormal BA levels to key pathologies in AD and PD (Mahmoudian Dehkordi et al., 2018; Yakhine-Diop et al., 2020; Zhang et al., 2022; Ma et al., 2024). However, the field is characterized by complexity and apparent contradictions. While certain BAs, such as tauroursodeoxycholic acid (TUDCA), show neuroprotective effects in preclinical models (Ahmed et al., 2022), others, such as deoxycholic acid (DCA), exacerbate neurotoxicity (Li et al., 2024b). Furthermore, some BAs appear to exert bidirectional mechanisms of action (Oleszycka et al., 2023; Qu et al., 2025). This “dual-role” nature of BAs underscores a key unresolved issue: the biological outcomes of BA signaling are context-dependent and contingent on the specific BA species, the host’s metabolic state, and the composition of the gut microbiota.

**Figure 2:**
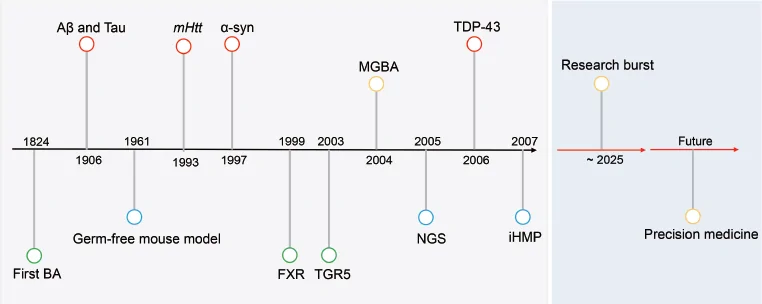
Timeline of converging milestones in neurodegeneration, microbiota, and BA research. This timeline illustrates the key conceptual and technological milestones that have shaped our current understanding of neurodegenerative diseases through the lens of the MGBA and BA signaling. The research field is divided into a foundational period (gray background) and a subsequent “explosion period” (blue background). The foundational period was characterized by the independent discovery of core components—including key pathogenic proteins (red), BAs and their receptors (green), as well as key technologies/models (blue), and key theories (yellow). The ongoing “explosion period,” catalyzed by the formalization of the MGBA concept and advances in sequencing and omics technologies, is an era of integration, in which these once-separate fields are converging. The red arrow symbolizes a forward-looking trajectory in which this new, integrated knowledge is expected to drive the future of neurodegenerative disease research toward precision medicine guided by the complex interplay of multiple factors. Aβ: Amyloid-β; BA: bile acid; FXR: farnesoid X receptor; iHMP: integrative human microbiome project; MGBA: microbiota-gut-brain axis; mHtt: mutant huntingtin protein; NGS: next-generation sequencing; TDP-43: TAR DNA-binding protein 43; TGR5: Takeda G protein-coupled receptor 5; α-syn: α-synuclein.

Despite this progress, important research gaps persist, hindering the therapeutic application of BAs. First, although the MGBA is widely recognized, many studies still treat the gut microbiota as a confounding factor rather than as a core mechanistic driver of the functional BA pool. Second, and most crucially, the impact of sexual dimorphism on BA metabolism and its implications for neurodegenerative diseases has been largely overlooked. This gap limits understanding of why these disorders often exhibit sex-specific prevalence and progression, and how this relates to gut-brain communication. To contextualize these research gaps and illustrate the convergence of previously disparate fields, we present a timeline of key milestones that have shaped current understanding (**Figure 2**).

Building on this conceptual framework, this review aims to address these gaps by providing an integrated synthesis of BA signaling in neurodegenerative diseases. The contribution of this review lies in its parallel and intertwined analysis of two central pillars: (1) the role of the gut microbiota as the primary engine of BA biotransformation and the interplay between BAs and the gut microbiota; (2) the role of sexual dimorphism as a regulator of the microbiota-BA-brain axis. By systematically linking BA metabolism to common pathological mechanisms from the perspectives of the microbiota and sex, we propose a conceptual framework for re-evaluating disease pathogenesis and designing more precise, BA-brain-oriented therapeutic strategies.

## Search Strategy

We conducted a comprehensive literature search of the PubMed database to identify studies relevant to this narrative review. The search was conducted up to August 2025. The search had no lower date limit to include foundational literature, but priority was given to recent publications to ensure the review reflects the current state of the field. Articles selected primarily consisted of original research (including preclinical and clinical studies) and review articles published in English. The criteria for selection were to first confirm relevance to the present article based on the title and abstract, followed by a full-text analysis. Search terms included various keywords, such as “bile acids” and various BA isoforms (e.g., DCA and TUDCA), “FXR,” “TGR5,” “neurodegenerative diseases,” “Alzheimer’s disease,” “Parkinson’s disease,” “amyotrophic lateral sclerosis,” “Huntington’s disease,” “multiple sclerosis,” “microbiota–gut–brain axis,” or “gut microbiota,” “sexual dimorphism,” and “sex.” We also conducted searches combining keywords, such as “bile acids AND neurodegenerative diseases,” “bile acids AND gut microbiota,” “bile acids AND sex,” and various other combinations (not all combinations are listed). After comprehensive screening, approximately 250 articles were included in the final review.

## Pathophysiological Landscape of Neurodegeneration: From Distinct Proteinopathies to Convergent Mechanisms

### Alzheimer’s disease

AD, the most common form of dementia (Kim et al., 2025; Liu et al., 2025), is a neurodegenerative disorder characterized by progressive cognitive impairment that ultimately leads to complete dependence (Ren et al., 2022). The primary pathological hallmarks of AD are extracellular plaques formed by amyloid-β (Aβ) deposition and intracellular neurofibrillary tangles (NFTs) composed of hyperphosphorylated tau (Graff-Radford et al., 2021). These proteinopathies arise from the abnormal metabolism of the amyloid precursor protein (APP) and the hyperphosphorylation of tau (Orobets and Karamyshev, 2023). The accumulation of Aβ, particularly its soluble oligomeric forms, is thought to initiate a pathological cascade that promotes tau pathology, neuroinflammation, oxidative stress, and synaptic dysfunction, creating a vicious cycle of neurodegeneration (Gallego-Rudolf et al., 2024).

Evidence indicates that the gut microbiota, as a key peripheral regulator, influences brain function and host behavior through the MGBA. Crucially, it also contributes significantly to the initial pathological mechanisms of AD (Zhang and Yan, 2023). Mechanistic studies further indicate that gut microbes directly or indirectly influence CNS function via diverse metabolites, including BAs (Hou et al., 2022). These findings provide new insights into AD pathogenesis and point to potential BA-targeted therapeutic strategies.

### Parkinson’s disease

PD is the second most common neurodegenerative disease, pathologically defined by the progressive loss of dopaminergic neurons in the substantia nigra and the formation of intracellular Lewy bodies (Pang et al., 2022). The core constituent of Lewy bodies is misfolded α-synuclein (α-syn), a presynaptic protein that aggregates due to a combination of genetic and environmental factors (Bloem et al., 2021). This aggregation impairs critical cellular processes, including the ubiquitin-proteasome and autophagy-lysosome systems, and induces mitochondrial dysfunction and oxidative stress, leading to a vicious cycle of neuronal death (Ben-Shlomo et al., 2024).

A recent study challenges the traditional brain-centric view of PD, with the “gut-brain propagation hypothesis” suggesting that pathology may originate in the enteric nervous system (ENS) and travel to the brain via the vagus nerve (Yang et al., 2024b). This is supported by evidence that gastrointestinal symptoms often precede motor deficits and that pathological α-syn aggregates are found in the gut long before appearing in the brain (Park et al., 2025). Alterations in gut microbiota composition also correlate with disease progression (Wang et al., 2025b). Collectively, this evidence positions the MGBA as a potential driver of PD initiation, underscoring the importance of gut-derived signals such as BAs in modulating disease susceptibility.

### Huntington’s disease

HD is an autosomal dominant neurodegenerative disorder characterized by motor, cognitive, and psychiatric symptoms (Bates et al., 2015). It is caused by an expansion of CAG trinucleotide repeats in the *HTT* gene, resulting in a mutant huntingtin protein (mHTT) with an abnormally long polyglutamine (polyQ) tract (Aviner et al., 2024). This mutant protein is prone to aggregation, primarily affecting medium spiny neurons in the striatum (Tshilenge et al., 2023; Shah et al., 2025). The toxicity of mHTT stems from its disruption of multiple cellular functions, including transcriptional regulation, protein clearance, mitochondrial function, and axonal transport, all of which contribute to neuroinflammation and neuronal death (Vitet et al., 2023; Shah et al., 2025).

Patients with HD often exhibit systemic energy dysmetabolism, including peripheral insulin resistance (e.g., elevated homeostatic model assessment of insulin resistance [HOMA-IR]), progressive weight loss despite adequate caloric intake, and increased resting energy expenditure (Campesan et al., 2023). In the brain, HD features reduced glucose utilization (especially in the striatum and cortex), mitochondrial dysfunction, and decreased adenosine triphosphate (ATP) production (Campesan et al., 2023). Moreover, alterations in the gut microbiota may contribute to energy dyshomeostasis and disease progression in HD, highlighting a potential role for the BA system (Ekwudo et al., 2025).

### Amyotrophic lateral sclerosis

ALS is a fatal neurodegenerative disease characterized by the progressive degeneration of upper and lower motor neurons, leading to muscle paralysis and respiratory failure (Feldman et al., 2022). Approximately 90% of ALS cases are sporadic (sALS), and the remaining 10% are familial (fALS) (Al Dera and AlQahtani, 2024). The pathology is complex, involving protein inclusions in motor neurons and glia, driven by mutations in genes such as *C9orf72*, *SOD1*, *TARDBP*, and *FUS* (Amin et al., 2020). The most common hallmark, present in **~**97% of cases, is the cytoplasmic mislocalization and aggregation of TAR DNA-binding protein 43 (TDP-43) (Grima et al., 2025). This proteinopathy, along with others, disrupts critical cellular processes and involves complex non-cell-autonomous mechanisms, including excitotoxicity and neuroinflammation driven by activated glia (Rubino et al., 2023).

Recent studies indicate that patients with ALS exhibit alterations in the gut microenvironment. These include microbiota dysbiosis, increased gut barrier permeability, and abnormal levels of microbial metabolites (Di Gioia et al., 2020; Guo et al., 2023). Analyses in patients and animal models show reduced short-chain fatty acid (SCFA)–producing bacteria and increased potentially toxigenic or pro-inflammatory taxa (Kaul et al., 2024). These changes correlate with disease progression rate and patient survival (Di Gioia et al., 2020). Such alterations may influence neuroinflammation and motor neuron survival via the MGBA. This offers new perspectives for understanding ALS pathogenesis and for exploring therapeutic strategies, such as modulating the gut microbiota or BA signaling.

### Convergent pathological pathways: A unifying framework

Despite differences in clinical presentation, predominant brain regions, and key pathogenic proteins, neurodegenerative diseases share fundamental pathological processes at the molecular and cellular levels.

A central feature is the dysregulation of protein homeostasis (proteostasis), observed across distinct proteinopathies: Aβ and tau in AD, α-syn in PD, mHTT in HD, and TDP-43 in ALS (Xu et al., 2025). Misfolded proteins form toxic oligomeric species and propagate between cells in a prion-like manner, triggering neuroinflammation and driving a self-amplifying cascade of neurodegeneration (Wang et al., 2021; Smethurst et al., 2022; Abidi et al., 2024; Todd et al., 2024). Neuroinflammation is another key driver of progression, characterized by the chronic activation of microglia and astrocytes, which release pro-inflammatory cytokines and contribute to a neurotoxic environment (Cuní-López et al., 2022; Boskovic et al., 2024). This process can also impair BBB integrity (Fornari Laurindo et al., 2024). Although inflammatory signatures differ across diseases in space, time, and molecular composition, chronic excessive neuroinflammation is a common feature, suggesting that targeting inflammatory pathways may have broad neuroprotective potential. Multiple sclerosis (MS), a paradigmatic disease bridging neuroinflammation and neurodegeneration, exemplifies this link (Meier et al., 2024; Szlufik et al., 2024). Notably, the BA system appears to regulate this inflammation-neurodegeneration axis, as BA metabolism is disturbed in MS patients, and BA-targeted interventions can alleviate disease in preclinical models by suppressing glial activation and promoting regulatory T cells (Jia et al., 2021b; Antonini Cencicchio et al., 2025). Further convergent pathways include energy dysmetabolism and mitochondrial dysfunction, leading to diminished ATP production and excess oxidative stress, and excitotoxicity, driven by excessive glutamate signaling and calcium overload (Neves et al., 2023; Yang et al., 2023; Wang et al., 2025c).

Finally, dysregulation of the MGBA is increasingly recognized as a common feature across neurodegenerative diseases. All discussed diseases exhibit gut microbiota dysbiosis. Typical changes include reduced microbial diversity, loss of beneficial taxa (e.g., SCFA producers), and relative increases in potentially harmful or pro-inflammatory bacteria (Liu et al., 2023; Di Chiano et al., 2024). Concurrently, gut-barrier integrity may be compromised (increased intestinal permeability or “leaky gut”), evidenced by downregulated expression of tight-junction proteins. This permits microbial components (e.g., lipopolysaccharide, LPS) and metabolites to enter the circulation, potentially triggering or exacerbating systemic and neuroinflammation (Kesika et al., 2021). Levels of key microbial metabolites (such as SCFAs and BAs) are also often altered; these molecules possess important signaling functions. Activation of the gut immune system and alterations in autonomic nervous system function (especially the vagus nerve) are also involved (Knox et al., 2023; Cao et al., 2024). Although disease-specific MGBA features differ, a common mechanism—disturbance of the gut microenvironment affecting brain health via neural, immune, endocrine, and metabolic pathways—appears conserved. These common MGBA changes suggest that the MGBA, as a bidirectional communication pathway, may be a central nexus linking the pathological processes of multiple neurodegenerative diseases.

Comparing these shared mechanisms deepens understanding of each disease and provides a basis for developing broadly applicable neuroprotective strategies. These include therapies aimed at enhancing protein clearance, targeting neuroinflammation, restoring mitochondrial function, or modulating the gut microenvironment. These commonalities help explain why the BA system may play a regulatory role across neurodegenerative diseases. As metabolic signaling molecules, BAs can influence multiple core processes, including inflammation, energy metabolism, gut microbiota composition, and neuronal survival. The BA system may thus serve as a crucial metabolic link between seemingly disparate neurodegenerative diseases. Subsequent sections detail how the BA system, by modulating these common pathological processes, may contribute to the pathogenesis and potential treatment of different neurodegenerative diseases.

## Bile Acid System: A Network of Multifunctional Signaling Molecules

### Bile acid metabolism: synthesis, microbial transformation, and circulation

BAs are 24-carbon sterols synthesized predominantly from cholesterol in the liver and are primary constituents of bile. They play crucial roles in lipid metabolism and in maintaining the integrity of the enterohepatic circulation. BAs are categorized as either primary or secondary, based on their origin.

**Figure 3:**
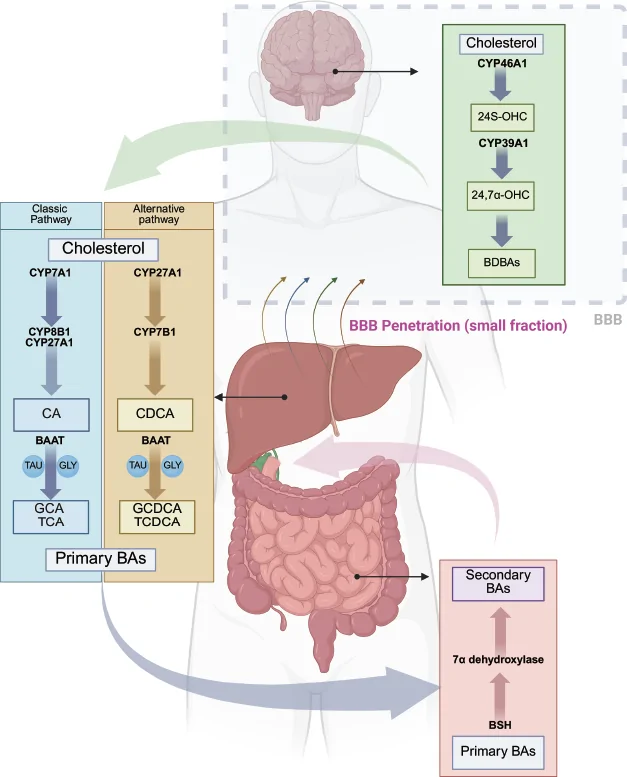
Synthesis and metabolism of BAs in brain–gut–liver axis. This diagram illustrates the synthesis and metabolism of BAs across the brain-gut-liver axis. In the brain, cholesterol is converted to BDBAs through 24S-OHC and 24,7α-OHC, catalyzed by CYP46A1 and CYP39A1. In primary BAs synthesis in the liver, the classic pathway converts cholesterol to CA via CYP7A1 and CYP8B1/CYP27A1, which then forms GCA and TCA with TAU and GLY. The alternative pathway converts cholesterol to CDCA via CYP27A1 and CYP7B1, forming GCDCA and TCDCA with TAU and GLY. The dotted box represents the BBB, and the four curved arrows of different colors (brown, blue, green, and red) represent a small number of BAs entering the brain through the BBB. In the gut, primary BAs are converted to secondary BAs by 7α dehydroxylase and bile salt hydrolases (BSH). A small fraction of BAs penetrates the BBB. 24,7α-OHC: 24,7α-hydroxycholesterol; 24S-OHC: 24S-hydroxycholesterol; BAAT: bile acid CoA: amino acid N-acyltransferase; BAs: bile acids; BDBAs: brain-derived bile acids; BBB: blood–brain barrier; BSH: bile salt hydrolase; CA: cholic acid; CDCA: chenodeoxycholic acid; CYP7A1: cholesterol 7-α-hydroxylase; CYP7B1: oxysterol 7-α-hydroxylase; CYP8B1: sterol 12-α-hydroxylase; CYP27A1: sterol 27-hydroxylase; CYP39A1: oxysterol 7-α-hydroxylase; CYP46A1: cholesterol 24-hydroxylase; GCA: glycocholic acid; GCDCA: glycochenodeoxycholic acid; GLY: glycine; TAU: taurine; TCA: taurocholic acid; TCDCA: taurochenodeoxycholic acid.

Primary BAs are synthesized in hepatocytes via enzymatic modifications of the sterol ring and side chain of cholesterol (Thomas et al., 2022). This synthesis occurs via two main pathways (**Figure 3**; Baptissart et al., 2013). The classic (neutral) pathway begins with the 7α-hydroxylation of cholesterol by cholesterol 7α-hydroxylase (CYP7A1). Subsequent reactions mediated by sterol 12α-hydroxylase (CYP8B1) and sterol 27-hydroxylase (CYP27A1) ultimately generate cholic acid (CA) and chenodeoxycholic acid (CDCA) (Russell, 2003). These two primary BAs can then be conjugated with glycine or taurine to form glycocholic acid (GCA), taurocholic acid (TCA), glycochenodeoxycholic acid (GCDCA), and taurochenodeoxycholic acid (TCDCA) (Hurley et al., 2022). Conjugation enhances the water solubility of BAs, facilitating their transport and function. Approximately 75% of BAs are synthesized via this pathway (Jia et al., 2021a).

In contrast to the classical pathway, BA synthesis can also proceed via an alternative route—often referred to as the “acidic” pathway. This process begins with the hydroxylation of cholesterol at the 27-position by CYP27A1, followed by an additional modification catalyzed by CYP7B1. In human hepatocytes, this pathway plays a relatively minor role, accounting for just under 10% of total BAs (TBAs) output (Duane and Javitt, 1999). Still, its contribution is far from negligible, especially under certain metabolic or pathological conditions. BA complexity extends beyond hepatic synthesis. Microbial transformations reshape BA physicochemical properties, receptor affinities, and signaling behaviors. Through processes such as deconjugation, dehydroxylation, and epimerization, the microbiota expands the signaling reach of BAs, enabling them to influence not only host metabolism and immune responses but also neural pathways. What begins as a set of hepatic metabolites becomes a dynamic repertoire of signaling molecules, some of which act on receptors such as FXR and TGR5 in tissues beyond the gut. In this sense, the microbiota serves not only as a downstream participant but also as a biochemical partner—fine-tuning BA function in ways the liver alone cannot achieve.

Microbial modification of BAs proceeds primarily via two mechanisms. First, deconjugation by BSHs—widely expressed in genera such as Bacteroides—removes glycine or taurine from primary BAs, generating unconjugated BAs and enabling subsequent transformations (Kiriyama and Nochi, 2023a). Second, 7α-dehydroxylation generates key secondary BAs such as DCA and lithocholic acid (LCA) (Kiriyama and Nochi, 2023a). Specific bacterial communities, represented by *Eggerthella lenta*, perform this critical biotransformation through their BA-inducible operon (bai operon) (Hylemon et al., 2018). These secondary BAs exhibit stronger receptor activation and signal transduction functions and thus may more profoundly affect host metabolism and immune responses (Guzior and Quinn, 2021).

Beyond these core reactions, additional genera—including *Romboutsia*, *Turicibacter*, *Blautia*, and *Ruminococcus*—modulate the BA pool by shaping secondary BA production, thereby influencing overall BA composition and intestinal homeostasis (Gadaleta et al., 2022). Thus, microbial modification not only determines the chemical composition of the BA pool but also expands BA signaling capacity. Microbiota-derived secondary BAs more effectively activate multiple host receptors, thereby regulating lipid metabolism, inflammatory responses, and intestinal barrier function (Collins et al., 2023). Conversely, shifts in the BA pool reshape microbial community structure, forming a bidirectional regulatory loop that helps maintain intestinal ecological balance and host health (Guo et al., 2022).

Once synthesized and microbially modified, these BAs—particularly in their conjugated forms, collectively known as bile salts—are stored in the gallbladder. Upon food intake, bile salts are released into the duodenum to facilitate the digestion and absorption of dietary lipids and fat-soluble vitamins (Ridlon et al., 2006). The majority of bile salts (approximately 95%) are reabsorbed in the terminal ileum and return to the liver to re-enter the bile, a process known as the enterohepatic circulation (Ridlon et al., 2006). Unabsorbed BAs pass into the colon, where they are further converted by microorganisms into secondary BAs. Some of these are passively absorbed into the bloodstream, while the remainder are excreted in the feces (Ridlon et al., 2006).

BAs exert regulatory functions *in vivo* through interactions with specific receptors. These receptors play roles in metabolic regulation and inflammatory responses. They also mediate BA effects in several physiological processes, including energy metabolism, homeostasis, and immune responses, underscoring the importance of BA-receptor signaling pathways.

### Key receptors mediating bile acid signaling

#### Nuclear receptor: Farnesoid X receptor

The nuclear receptor FXR is a ligand-activated transcription factor with predominant activity in the liver and intestine. In most mammals, two forms are present: FXRα and FXRβ. In humans, however, FXRβ is widely regarded as a nonfunctional pseudogene (Jiang et al., 2021). Thus, FXRα is the functional receptor. Through alternative splicing, a single *FXRα* gene gives rise to at least four distinct protein isoforms (α1–α4). Each variant exhibits distinct functions and tissue preferences (Boesjes et al., 2014). For instance, FXRα1 and α2 are found in high concentrations in the ileum and adrenal glands, while FXRα3 and α4 are present in the ileum and additionally expressed in the kidney (Jiang et al., 2021). The diversity in form and function underscores the context-specific nature of FXR signaling.

The best-known role of FXR is maintaining metabolic balance in peripheral tissues by regulating BA dynamics. It regulates the BA lifecycle, from synthesis and transport to enterohepatic circulation (Wang et al., 2024a). Mechanistically, upon ligand binding, FXR typically heterodimerizes with RXR, and this complex directly represses the transcription of CYP7A1, the rate-limiting enzyme for BA synthesis. Ileal FXR activation induces fibroblast growth factor 15/19 (FGF19 in humans), which circulates to the liver. There, it activates fibroblast growth factor receptor 4 (FGFR4) with the co-receptor β-Klotho (Li et al., 2024c). This triggers ERK/MAPK and p38/MAPK cascades, suppressing CYP7A1 transcription and promoting expression of BA-metabolism genes such as small heterodimer partner (*SHP*) (Song et al., 2023; Yu Cai Lim and Kiat Ho, 2024). This constitutes a negative feedback loop that prevents excessive BA synthesis. Conversely, disrupting FGFR4 signaling (e.g., with inhibitors) upregulates CYP7A1 and increases BA production (Yu Cai Lim and Kiat Ho, 2024).

By constraining BA synthesis, FXR also influences cholesterol homeostasis. By repressing CYP7A1, FXR regulates conversion of cholesterol to BAs, a key node in lipid homeostasis (Bao et al., 2022). FXR may influence HDL biogenesis and cholesterol efflux, potentially via the miR-144–ABCA1 pathway, though mechanisms remain incompletely defined (Goodwin et al., 2000). The influence of FXR also extends to triglycerides (TGs). Activation suppresses hepatic TG accumulation and reduces very-low-density lipoprotein secretion, lowering circulating TGs. This process is thought to occur via repression of SREBP-1c and its targets, likely involving small heterodimer partner and liver X receptors (LXRα/β) (Watanabe et al., 2004). Beyond metabolism, FXR exerts anti-inflammatory effects and supports epithelial barrier integrity. It antagonizes nuclear factor κ-light-chain-enhancer of activated B cells (NF-κB)-mediated signaling, thereby limiting pro-inflammatory cytokine production and maintaining intestinal homeostasis, particularly under stress (Kriaa et al., 2022). Collectively, these roles position FXR as a key integrator of metabolic, immune, and epithelial responses. Dysfunction in FXR signaling could be linked to metabolic and neuroinflammatory disorders.

#### Membrane receptor: Takeda G protein-coupled receptor 5

TGR5 is a G-protein-coupled receptor activated by BAs, with LCA and DCA among the more potent endogenous agonists, although the effects of DCA are comparatively weaker (Perino et al., 2021). TGR5 is broadly expressed in metabolically active tissues, including liver, gallbladder, intestine, kidney, spleen, skeletal muscle, brown adipose tissue, and brain, in rodents and humans (Keitel and Dieter, 2012; Lun et al., 2024). Such extensive distribution enables BAs to modulate diverse cellular functions across hepatocytes, enterocytes, immune cells, and neurons. BA–TGR5 interactions initiate downstream cascades that regulate secretion, metabolism, inflammatory responses, and neural transmission (Shapiro et al., 2018). This establishes TGR5 as a mediator of BA effects.

**Figure 4:**
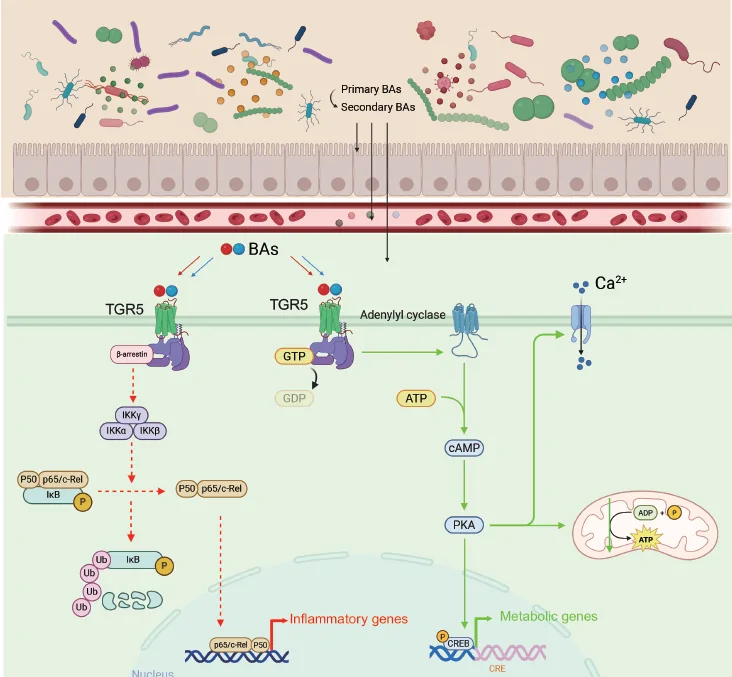
Schematic overview of the two canonical TGR5 signaling pathways. The overall BA pool that reaches the cell is a complex mixture originating from both host synthesis (primary BAs) and microbial transformation (secondary BAs), as depicted in the upper panel (yellow background). (Left panel) β-Arrestin-dependent anti-inflammatory pathway: Upon BA binding, TGR5 can recruit β-arrestin, which in turn inhibits the IKK complex (IKKα/β/γ). This prevents the phosphorylation and subsequent ubiquitination-dependent degradation of IκB, effectively keeping the NF-κB (p65/p50) complex sequestered in the cytoplasm and suppressing the transcription of pro-inflammatory genes. (Right panel) Gs protein-dependent metabolic and neuroprotective pathway: Alternatively, TGR5 activation can trigger a Gs protein-dependent cascade. This leads to the activation of adenylyl cyclase, an increase in intracellular cAMP levels, and subsequent activation of PKA. PKA can then phosphorylate the transcription factor CREB, promoting the expression of metabolic and neurotrophic genes. This pathway is also linked to the enhancement of mitochondrial ATP production and the modulation of intracellular Ca²⁺ signaling. The red dashed arrows represent the inhibitory pathway, and the green solid arrows represent the activating pathway. It is crucial to note that both pathways can be activated by various BA ligands, but the ultimate biological outcome is highly context-dependent. The BA pool consists of hydrophobic BAs (blue), which are potent endogenous agonists but can be cytotoxic at high concentrations, and hydrophilic BAs (red), which are primarily protective and used therapeutically. Currently, there is insufficient evidence to suggest that these different BA classes strictly segregate their signaling to one pathway over the other. Therefore, the net effect of TGR5 activation in neurodegeneration is determined not only by these signaling pathways but also by the overall balance of the BA pool and the specific pathological state of the cell. ADP: Adenosine diphosphate; ATP: adenosine triphosphate; BAs: bile acids; cAMP: cyclic adenosine monophosphate; CRE: cAMP response element; CREB: cAMP response element-binding protein; GDP: guanosine diphosphate; GTP: guanosine triphosphate; IκB: inhibitor of NF-κB; IKK: IκB kinase; NF-κB: nuclear κB; PKA: protein kinase A; TGR5: G protein-coupled bile acid receptor 5; Ub: ubiquitin.

Activation of TGR5 increases intracellular cAMP, which in turn activates PKA (**Figure 4**) and initiates a cascade of downstream effects. These include enhanced mitochondrial oxidative phosphorylation and, in some contexts, calcium-channel opening that raises cytosolic Ca^2+^ levels (Darmanto et al., 2025). A key outcome is stimulation of glucagon-like peptide-1 release from intestinal L cells. This affects glycemic control, appetite, and overall energy homeostasis (Thomas et al., 2009). In adipose tissue, TGR5 contributes to energy balance. It upregulates uncoupling protein-1 to promote thermogenesis, with relevance to obesity and insulin resistance (Chen et al., 2023a). Thus, TGR5 functions as a hub for glucose control, energy use, and nutrient sensing. Its functions extend to lipid metabolism. It can influence hepatic BA synthesis via pathways involving CYP7A1, integrating metabolic sensing with BA feedback. In the heart, impaired TGR5 signaling has been linked to lipid accumulation and metabolic dysfunction. This suggests a broader role for TGR5 in cardiometabolic health (Wang et al., 2024b, 2025c).

TGR5 also regulates immunity, mainly by modulating the NF-κB signaling axis (**Figure 4**). Under basal conditions, NF-κB (typically a p65/p50 dimer) is retained in the cytoplasm by IκB. Inflammatory stimuli such as LPS or TNF-α promote IκB phosphorylation and degradation. This enables NF-κB nuclear translocation and transcription of pro-inflammatory genes. TGR5 modulates this step. It stabilizes IκBα by enhancing its interaction with β-arrestin 2, limiting IκBα phosphorylation and preventing p65 nuclear entry (Wang et al., 2011). By restraining NF-κB activation, TGR5 shifts immune responses toward a less inflammatory profile. This reduces expression of cytokines such as IL-6 and TNF-α and may upregulate anti-inflammatory mediators such as A20 (Li et al., 2024a; Wang et al., 2025a). Beyond innate immunity, TGR5 also influences adaptive immunity by altering the behavior of immune-cell subsets (Fiorucci et al., 2024).

TGR5 has broad physiological relevance across organ systems. Its effects are prominent in the gastrointestinal tract. For instance, it helps maintain BA circulatory balance by promoting gallbladder contraction and bile release (Lange et al., 2024). It also strengthens the intestinal barrier and reduces endotoxin leakage, potentially via Hippo/YAP signaling (Qiang et al., 2025). In the liver, TGR5 activation exerts hepatoprotective effects by attenuating inflammation and oxidative stress in models of drug-induced and alcoholic liver injury (Biagioli et al., 2023). Similar benefits are reported in metabolic liver disease, such as nonalcoholic steatohepatitis. Here, TGR5 reduces lipid accumulation and improves insulin resistance, indicating therapeutic potential (Zhang et al., 2025a). Its influence also extends to the cardiovascular and renal systems. In the heart, TGR5 optimizes lipid metabolism and mitochondrial function, which may confer cardioprotection. In the kidney, it supports blood pressure homeostasis by modulating sodium reabsorption, principally via the epithelial sodium channel (Wang et al., 2024b; Li et al., 2025a). A recent study points to an emerging role for TGR5 in tumor immunology (Chen et al., 2024). TGR5 may disrupt immune escape by interfering with STAT3/PD-L1 signaling (Chen et al., 2024).

Although often considered a peripheral player, TGR5 is increasingly recognized for its roles in the CNS. It appears to contribute to neural and behavioral homeostasis. A major component is its influence on central energy regulation and feeding behavior. This is evident in neurocircuits such as hypothalamic neurons that respond to BA signals, which can affect food intake, glucose dynamics, and overall metabolic state (Zizzari et al., 2024). TGR5 is expressed in GABAergic neurons of the lateral hypothalamic area. It modulates their excitability, which may help fine-tune responses to internal metabolic cues (Li et al., 2024d). The role of TGR5 in the CNS is not limited to neurons. It also contributes to neuroimmune regulation. It shapes microglial and astrocytic activity, thereby influencing CNS inflammatory tone and helping preserve immune balance (Darmanto et al., 2025). To consolidate these networks, we summarize key molecular pathways downstream of TGR5 and their principal biological effects in the CNS in **Additional Table 1**. This table compares how TGR5 activation translates into distinct outcomes, from anti-inflammatory and neuroprotective actions to regulation of cellular metabolism and autophagy.

**Additional Table 1:** Key downstream signaling pathways of TGR5 in the CNS

Classification	Pathway	Core molecular cascade	Main biological effect	Reference
Anti-inflammatory pathways	Pellino3-caspase-8-NLRP3	TGR5 activation → Pellino3 ↑ → caspase-8 inhibition → suppression of NLRP3 inflammasome assembly	Anti-inflammatory; reduces infarct volume and neuronal injury	Liang et al., 2021
	cAMP/PKA/CREB	TGR5 → Gs protein → cAMP ↑ → PKA ↑ → p-CREB ↑	Anti-inflammatory (↓TNF-α, IL-6, IL-1β); inhibits microglial activation; improves cognition	Jin et al., 2021
	cAMP/PKA/NLRP3 inflammasome	TGR5 ↑ cAMP ↑ → PKA ↑ → suppression of NLRP3- ASC inflammasome activation	Anti-inflammatory; reduces brain edema and neuronal injury	Hu et al., 2021
	Akt/NF-KB	TGR5 → suppression of p-Akt, p-IkBα/p-NF-KB	Anti-inflammatory (↓TNF-α, IL-6, IL-1β); inhibits glial inflammation	Zhu et al., 2022
Neuroprotective pathways	cAMP/PKA/CREB-BDNF	TGR5 ↑ cAMP ↑ → PKA ↑ → p-CREB ↑ → BDNF ↑	Anti-inflammatory, anti-apoptotic; promotes neuroprotection and synaptic plasticity; improves memory	Wu et al., 2019b
	EGFR/ERK	TGR5 → EGFR transactivation → ERK ↑	Regulates excitability of hippocampal CA3 neurons; potentially modulates learning and memory	Romero-Ramirez and Mey, 2024
Autophagy & mitochondrial regulation	AMPK/mTOR autophagy	TGR5 → ↑ p-AMPK, ↓ p-mTOR → ↑ LC3II/I, ↑ Beclin1, ↓ p62	Enhances autophagy, reduces inflammation, protects neurons	Ni et al., 2025
	Pinkl/Parkin mitophagy	TGR5 → ↑ Pink1, ↑ Parkin	Promotes mitophagy; improves mitochondrial function and antioxidative defense	Ni et al., 2025
Metabolic regulation pathways	TGR5-Leptin/STAT3	TGR5 in NTS → enhances LeptinR-STAT3 signaling	Suppresses hyperphagia	Bruce et al., 2025
	TGR5-AgRP/NPY	TGR5 activation in arcuate nucleus AgRP neurons → ↓ AgRP/NPY expression	Suppresses hunger signaling; induces satiety/ anorexigenic behavior	Romero-Ramirez and Mey, 2024

AgRP: Agouti-related peptide; Akt: protein kinase B; AMPK: AMP-activated protein kinase; ASC: apoptosis-associated speck-like protein containing a CARD; BDNF: brain-derived neurotrophic factor; cAMP: cyclic adenosine monophosphate; CREB: cAMP response element-binding protein; EGFR: epidermal growth factor receptor; ERK: extracellular signal-regulated kinase; Gs: stimulatory G protein; IL-1β: interleukin-1β; IL-6: interleukin-6; IkBα: inhibitor of KBα; LC3: microtubule-associated protein 1A/1B-light chain 3; LeptinR: leptin receptor; mTOR: mechanistic target of rapamycin; NF-kB: nuclear factor K-light-chain-enhancer of activated B cells; NLRP3: NLR family pyrin domain containing 3; NPY: neuropeptide Y; NTS: nucleus of the solitary tract; p62: sequestosome-1; Pinkl: PTEN-induced kinase 1; PKA: protein kinase A; STAT3: signal transducer and activator of transcription 3; TGR5: G protein-coupled bile acid receptor 5; TNF-α: tumor necrosis factor-α.

Beyond FXR and TGR5, additional receptors respond to specific BAs, including pregnane X receptor (PXR), vitamin D receptor (VDR), constitutive androstane receptor (CAR), and muscarinic acetylcholine receptor M2 (M2 mAChR). These receptors modulate diverse physiological pathways, such as xenobiotic metabolism, BA homeostasis, and signal transduction, across multiple tissues (**Additional Table 2**; Huang et al., 2023). They are detected in brain tissue, but their BA interactions and potential neuroprotective roles remain incompletely characterized (Huang et al., 2023; Colón Ortiz et al., 2024). Although their cerebral presence suggests involvement in intracerebral BA metabolism and signaling, the precise mechanisms of response to BAs remain to be defined.

**Additional Table 2:** Characteristics of additional BA receptors

Receptor	Type	BA ligand	Tissue distribution	Physiological function	Associated disease	Reference
VDR	NR	LCA and its derivatives	Liver, stomach, intestine, immune cells	Regulation of BA metabolism, immunoregulation	IBD, CD, hepatitis	Fiorucci et al., 2020; Thibaut and Bindels, 2022
PXR	NR	LCA, DCA	Liver, kidney, lung	Liver protection, regulation of inflammation	IBD, CD	Huang et al., 2018; Wilson et al., 2020
CAR	NR	Secondary BAs	Liver, Intestine, Immune cells	Defense against BA toxicity, regulation of metabolism-related genes	CD, Cholestasis	Guo et al., 2003; Chen et al., 2021
RORγt	NR	LCA and its derivatives	Immune cells	Immunoregulation	Immune system disease	Thibaut and Bindels, 2022
LXR	NR	HDCA	Liver, intestines, fat tissue	Cholesterol metabolism, immunoregulation	NAFLD, HCC	Paul et al., 2022; Rezen et al., 2022
S1PR2	MR	Conjugated BAs	Liver, colon	Reduce liver inflammation, regulate vascular permeability, immune cell transport	Cholestasis, inflammatory liver disease, fatty liver disease	Kwong et al., 2015
α5β1 Integrin	MR	TUDCA	Epithelial cells, fibroblasts, cancer cells	Cell adhesion, migration, proliferation, activation of NTCP transporters in hepatocytes	Fibrosis, cancer metastasis therapy, metabolic disease	Rosa et al., 2021; Janus-Bell and Mangin, 2023
M3R	MR	Taurine conjugated BAs	CNS, gastrointestinal tract, smooth muscle	Signal transduction	CRC, PDAC	Tolaymat et al., 2019; Nenkov et al., 2023

BA: Bile acid; CAR: constitutive androstane receptor; CD: Crohn's disease; CNS: central nervous system; CRC: colorectal cancer; DCA: deoxycholic acid; HCC: hepatocellular carcinoma; HDCA: hyodeoxycholic acid; IBD: inflammatory bowel disease; LCA: lithocholic acid; LXR: liver X receptor; M3R: muscarinic acetylcholine receptor M3; MR: membrane receptor; NAFLD: non-alcoholic fatty liver disease; NR: nuclear receptor; NTCP: sodium/taurocholate cotransporting polypeptide; PDAC: pancreatic ductal adenocarcinoma; PXR: pregnane X receptor; RORyt: retinoid-related orphan receptor γt; S1PR2: sphingosine-1-phosphate receptor 2; TUDCA: tauroursodeoxycholic acid; VDR: vitamin D receptor.

### Bile acid communication with the central nervous system: Peripheral and brain-derived pathways

Brain-derived bile acids (BDBAs) and peripheral bile acids (PBAs) act through distinct mechanisms in the brain, and their origins and regulatory pathways differ. The brain, which has the highest cholesterol content of any organ, relies on cholesterol for the formation of cell membranes, myelin sheaths, synapses, and dendrites (Björkhem and Meaney, 2004). Because the BBB limits cholesterol exchange with the circulation, excess brain cholesterol is partly cleared via pathways that include conversion to BA precursors (**Figure 3**; Xing et al., 2023). The brain maintains a relatively independent cholesterol metabolism system in which BDBA synthesis and regulation are central. To maintain homeostasis, neurons convert excess cholesterol into 24S-hydroxycholesterol (24S-OHC) via CYP46A1, which is expressed almost exclusively in the brain (Björkhem et al., 2001). Most 24S-OHC crosses the BBB and enters the systemic circulation for hepatic metabolism. Conversely, a portion undergoes 7α-hydroxylation in the brain by enzymes such as CYP39A1, forming 24,7α-dihydroxycholesterol, a step in BDBA synthesis (Lund et al., 1999; Björkhem et al., 2001). Recent high-sensitivity mass-spectrometry studies challenge the view that brain BAs are merely peripheral spillover. A recent study provided evidence that the rodent brain contains its own BA pool that is region-specific (Reuter et al., 2025). For example, BA profiles in the cortex and hippocampus differ from those in the cerebellum, implying local regulation (Reuter et al., 2025). These findings indicate that the brain has an independent, regionalized BA network rather than being a passive recipient.

BDBAs can engage intracerebral receptors such as FXR and TGR5. They can modulate neurotransmitter release, neuroinflammatory tone, and synaptic plasticity (Xing et al., 2023; Sabahat et al., 2024). These metabolites can alter dopamine transporter activity, directly affecting neuronal signal propagation. One molecule of interest is 24S-OHC. As a product of brain-derived cholesterol metabolism, 24S-OHC functions as a neuromodulator. First, it directly regulates neurotransmitter release and synaptic transmission. At the neuromuscular junction, 24S-OHC can inhibit synaptic-vesicle recycling, thereby reducing neurotransmitter release (Kasimov et al., 2017; Mukhutdinova et al., 2018). Second, it exerts dual effects on N-methyl-D-aspartate (NMDA) receptors. Under physiological conditions, 24S-OHC is a positive allosteric modulator of NMDA receptors. This enhances receptor-mediated calcium influx and electrophysiological activity, which is important for learning and memory (Sun et al., 2024). However, under pathological conditions (e.g., ischemia or hypoxia), this enhancement may exacerbate glutamatergic excitotoxicity, promoting calcium overload and neuronal damage (Sun et al., 2017). In addition, 24S-OHC broadly affects neuronal function by interacting with ion channels and nuclear receptors. For example, it inhibits large-conductance calcium-activated potassium channels (BK channels), affecting neuronal excitability and action-potential patterns (Wang et al., 2022). These functions highlight the neuromodulatory role of the brain’s metabolic axis, which may be involved in neurodegenerative progression. This is particularly evident in the brain’s cholesterol-turnover system, governed by the neuron-enriched enzyme CYP46A1 (Koike et al., 2021). This enzyme is predominantly expressed in neurons, the primary site of brain cholesterol storage, and converts excess cholesterol into transportable 24S-OHC (Giannelli et al., 2025). This neuron-centric role links its function to neuronal health (Koike et al., 2021). In AD, decreased CYP46A1 activity and dysregulated 24S-OHC levels are associated with neuronal injury and cognitive decline (Shu et al., 2024). This metabolically driven dysfunction can occur independently of microbial influence. Thus, BDBAs may contribute intrinsically to neurodegenerative pathology under certain conditions (Monteiro-Cardoso et al., 2021). Although the role of 24S-OHC as the starting point for BDBA synthesis is relatively well established, research on the types, concentrations, distribution, and functions of downstream brain BAs remains limited. Mechanistic understanding of BDBA biology still relies largely on indirect inferences about their functions. Therefore, systematically characterizing the brain BA repertoire, distinguishing exogenous from endogenous species, and elucidating BDBA roles under physiological and pathological conditions are important goals for future research.

In contrast to BDBAs, PBAs originate in the liver from cholesterol catabolism, and their primary actions occur in the intestine. A portion bypasses reabsorption and enters the systemic circulation via the portal vein. A small subset then accesses the CNS. They cross the BBB via passive diffusion or, in some cases, active transport (Mahmoudian Dehkordi et al., 2018). BBB permeability depends on molecular features. For example, more hydrophobic BAs such as LCA and DCA show greater BBB permeability (Shibuya et al., 2024). Earlier observations reported only trace amounts of unconjugated BAs in rodent brains. A subsequent study identified a broader spectrum of BA species in brain tissue, including conjugated derivatives (Perino et al., 2021). These compounds may act as neuroactive steroids. A study reports inhibition of neuronal apoptosis, among other effects (Griffiths et al., 2019).

PBA entry into the brain is not uniformly beneficial. Certain hydrophobic PBAs can actually be neurotoxic. Mechanisms may include compromising BBB integrity and facilitating Aβ plaque formation, potentially via enhanced docosahexaenoic-acid oxidation (Li et al., 2024b). Once in the brain, PBAs can engage receptors such as TGR5, influencing neuronal metabolism and neuroinflammatory signaling (Li et al., 2024b; Monteiro-Cardoso et al., 2024). Both BDBAs and PBAs exert diverse regulatory functions. They act primarily through receptor-mediated modulation. FXR and TGR5 are central receptors. They function as nodes in networks governing metabolism, immune responses, energy balance, and homeostasis. Collectively, these findings suggest that neural regulation cannot be fully understood without considering BA-related signaling pathways.

Although BDBAs and PBAs have distinct origins and biosynthetic routes, they do not operate in isolation within the CNS. They likely interact through layered, sometimes synergistic, mechanisms. They converge on shared receptors. Both classes can activate nuclear and membrane receptors such as FXR and TGR5. However, outcomes differ. Responses vary with BA subtype, concentration, and the local cellular environment. Dynamics range from additive signaling to antagonistic feedback. Outcomes depend on receptor occupancy and pathway cross-talk. Through these receptors, BAs can influence both cerebral metabolism and immune tone. However, nuances remain. Receptor responsiveness often reflects BA structure and origin. For example, CDCA appears protective, dampening excitotoxicity by inhibiting NMDA receptors. Conversely, a hydrophobic BA such as LCA can be neurotoxic, possibly via opposite effects at similar molecular targets. This functional divergence underscores the pleiotropic nature of BA receptors. This indicates that identical receptors can produce markedly different responses depending on ligand properties (Ehtezazi et al., 2023; Monteiro-Cardoso et al., 2024).

Metabolic interactions between the microbiota and the host are also relevant. In the periphery, bacteria convert primary BAs into secondary metabolites such as DCA and LCA, which can later cross into the CNS. These secondary BAs are important because they contribute to gut–brain signaling and can modulate neurological functions, including anxiety-related behaviors (Chen et al., 2023b). By contrast, BDBAs arise from pathways tightly linked to cholesterol turnover and clearance within the brain. As products of the brain’s metabolic network, they participate in local control of glucose and lipid dynamics. Together, this constitutes a dual-tier regulatory system. BDBAs operate locally within the brain, whereas PBAs can enter via systemic routes. This system may support metabolic homeostasis in the brain (Hurley et al., 2022; Xing et al., 2023).

In pathological contexts, the balance and interplay of these two BA classes influence disease progression. In neurodegenerative diseases, they may synergistically exacerbate or alleviate pathological processes. For example, certain peripheral hydrophobic BAs can promote neurotoxicity, whereas specific BDBAs (e.g., CDCA) or neuroprotective BAs entering the brain may reduce stroke infarct volume or inhibit neuronal death (Griffiths et al., 2019; Monteiro-Cardoso et al., 2024; Shibuya et al., 2024). Further research indicates that BA dyshomeostasis, such as an imbalanced intracerebral-to-peripheral ratio or abnormal levels of specific species, is associated with neurological disease progression (Yeo et al., 2023). This suggests that targeted pharmacological interventions in the signaling networks of these BA classes may offer novel therapeutic strategies for neurological disease (Hurley et al., 2022). Indeed, more evidence points to a characteristic BA dysregulation in AD. Findings include decreased neuroprotective primary BAs such as CDCA in the brain and increased neurotoxic, microbially derived secondary BAs such as LCA in peripheral circulation and the CNS (Baloni et al., 2020; Zhang et al., 2025b). This imbalance—the loss of a protective agent and the gain of a toxic one—is proposed to contribute to neuroinflammation, neuronal loss, and cognitive decline in AD (Baloni et al., 2020).

## Bile Acid Dysregulation in Neurodegenerative Diseases

### Bile acids and Alzheimer’s disease

An increasing number of studies indicate that BAs function not only as digestive agents but also as signaling molecules in the CNS, often acting through the MGBA (Huang et al., 2022; Wu et al., 2024). Clinical and animal studies report reduced serum primary BA levels and elevated secondary BA levels in patients with AD, suggesting a link between gut microbiota metabolism and cognitive decline (Mahmoudian Dehkordi et al., 2018; Ma et al., 2024). Notably, TCA levels are reduced in the CSF of patients with AD. These changes have increased recognition of BAs as potential biomarkers. Variations in BA concentration and composition may indicate AD pathology and inform early diagnosis (**Additional Table 3**). Analyses of brain tissue provide more direct evidence (Reuter et al., 2025). Critically, this dysregulation was highly region-specific and correlated with known AD pathology; for instance, several BAs such as TCA and TMCA were elevated in the cortex and hippocampus (Reuter et al., 2025). These findings suggest that BA accumulation in affected regions reflects active local dysmetabolism rather than merely systemic changes.

**Additional Table 3:** Association between BAs and AD biomarkers

Biomarker	Serum (Mahmoudian Dehkordi et al., 2018; Nabizadeh et al., 2024)	CSF (Andújar et al., 2024)
CSF Aβ	HDCA-1	
	DCA:CA-1	DCA1, Nor-DCA1
	TDCA:CA-1	GDCA1, GCDCA1
	GDCA:CA-1	7-KETOLCA1
	GLCA:CDCA-1	TDCA: DCA-1
	GLCA:GDCA-1	
CSF t-Tau	GCDCA1	
	GDCA1, HDCA1	TUDCA1, GDCA1
	TLCA1, GLCA1	GDCA: DCA1
	DCA:CA1	
	GLCA:CDCA1	
CSF p-Tau	GCDCA1, HDCA1	
	TLCA1, GLCA1	TUDCA-1, GDCA-1
	DCA:CA1	GDCA: DCA-1
	GLCA:CDCA1	
FDG-PET	GCDCA-1	
	TLCA-1, GLCA-1	
	GLCA:CDCA-1	
	GDCA:CA-1	
	TDCA:CA-1	
Aβ-PET	GDCA1	
	7-KETOLCA-1	
Tau-PET	GDCA1, TDCA1	
	DCA:CA1	

A value of "1" represents a significant positive correlation, while "-1" represents a significant negative correlation. 7-KETOLCA: 7-Ketolithocholic acid; Aβ: amyloid-β; CA: cholic acid; CDCA: chenodeoxycholic acid; CSF: cerebrospinal fluid; DCA: deoxycholic acid; FDG-PET: fluorodeoxyglucose positron emission tomography; GCDCA: glycochenodeoxycholic acid; GDCA: glycodeoxycholic acid; GLCA: glycolithocholic acid; HDCA: hyodeoxycholic acid; Nor-DCA: nor-deoxycholic acid; p-Tau: phosphorylated tau; TDCA: taurodeoxycholic acid; TLCA: taurolithocholic acid; t-Tau: total tau; TUDCA: tauroursodeoxycholic acid.

Links between BAs and AD are complex. BAs can influence AD pathology and clinical features through various mechanisms. Because BA species are diverse, the understanding of species-specific actions remains limited. Experimental and animal studies provide preliminary clues. Several BAs may mitigate AD-related pathology (Joo et al., 2003; Ochiai et al., 2021). Ursodeoxycholic acid (UDCA) may reduce neuroinflammation by inhibiting interleukin-1β and nitric oxide production in microglia (Joo et al., 2003). The endogenous BA TUDCA appears neuroprotective (Ahmed et al., 2022). It reduces Aβ deposition in AD mouse brains and improves memory deficits (Ochiai et al., 2021). TUDCA also mitigates LPS-induced cognitive impairment and neurotoxicity, likely by reversing neuroinflammation via NF-κB signaling (Wu et al., 2019a; Zangerolamo et al., 2021). Although receptor interactions are complex, this anti-inflammatory action resembles TGR5-mediated suppression of NF-κB. CDCA, a primary BA, is a potent endogenous FXR agonist. It enhances hippocampal insulin sensitivity and improves cognitive function in AD model rats. Mechanistically, CDCA has several reported actions. For example, CDCA reduces β-site APP-cleaving enzyme 1 expression in the hippocampus, decreases phosphorylation of insulin receptor substrate-1 at serine 307, increases activation of Akt, and induces CREB, which is associated with learning and memory (Bazzari et al., 2019). These neuroprotective outcomes are consistent with the roles of FXR in metabolic homeostasis and inflammation. This suggests a mechanistic link between CDCA, FXR, and therapeutic potential in AD.

### Bile acids and Parkinson’s disease

The relationship between PD and BAs is complex, involving disease mechanisms and potential treatments. Research indicates that dysregulated BA metabolism is implicated in PD onset and progression, with patients showing elevated plasma levels of unconjugated BAs such as CA, DCA, and LCA. Abnormal BA metabolism may exacerbate neurodegeneration (Yakhine-Diop et al., 2020; Zhang et al., 2022). A potential link is BA-mediated regulation of α-syn homeostasis through the MGBA. In enteric neurons, secondary BAs such as hyodeoxycholic acid (HDCA) may enhance α-syn clearance by activating autophagy pathways. This may slow PD progression (Kong et al., 2025). This concept has inspired therapeutic exploration. Both UDCA and its taurine conjugate, TUDCA, show significant efficacy in animal models of PD. They appear to inhibit glial activation, improve function, and reduce dopaminergic-neuron apoptosis (Luxenburger et al., 2022; Ni et al., 2025; Razavi et al., 2025). Moreover, a clinical trial of UDCA showed targeted restoration of mitochondrial function in patients with PD (Payne et al., 2020).

Although receptor activation can be beneficial, BA signaling in PD is complex. TGR5 illustrates this complexity. Both LCA and DCA are endogenous TGR5 agonists, but they can have opposite effects. LCA accelerates α-syn aggregation and increases cytotoxicity, whereas DCA inhibits fibril formation and reduces neurotoxicity (Kaur et al., 2024). This underscores that activating the same receptor with different ligands or in different cellular contexts can yield divergent outcomes. By contrast, activating FXR appears more consistently beneficial. CDCA, a primary BA and FXR agonist, alleviates motor dysfunction in MPTP-induced PD models. Mechanisms involve anti-inflammatory and antioxidant effects (Mehreen et al., 2025). This aligns with FXR’s role in suppressing neuroinflammation, linking CDCA, FXR, and therapeutic potential in PD. This complexity is opening new therapeutic directions, such as the development of C-23-modified BA derivatives, which show stronger mitochondrial-enhancing effects than UDCA in fibroblasts from patients with LRRK2-mutant PD (Luxenburger et al., 2022). The gut microbiota-BA interplay is a major regulatory pathway in PD. Alterations in BA and microbiota profiles after cholecystectomy may increase PD risk (Kim et al., 2021).

### Bile acids and Huntington’s disease

HD has been linked to metabolic dysregulation, with BA-metabolism abnormalities potentially contributing (Chiang et al., 2024; Gray et al., 2024). Studies report disrupted metabolism of BAs and their precursor cholesterol in patients with HD (Leoni et al., 2011; Chiang et al., 2024). For example, GCDCA and glycoursodeoxycholic acid (GUDCA) are elevated in symptomatic patients, and signs of dysmetabolism are present in presymptomatic carriers. This suggests that BA imbalance may precede symptoms and contribute to disease progression (Chiang et al., 2024). Notably, levels of the brain cholesterol metabolite 24S-OHC, a precursor in brain-derived BA synthesis, are reduced in patients with HD. As a positive allosteric modulator of NMDA receptors, its deficiency may exacerbate HD-related glutamatergic dysfunction (Gray et al., 2024).

Systemic TUDCA in R6/2 mice reduced striatal atrophy and apoptosis, inhibited huntingtin inclusions, and improved motor and sensorimotor deficits (Keene et al., 2002). This is consistent with evidence that other hydrophilic BAs, such as UDCA and GUDCA, show therapeutic potential in HD models (Huang et al., 2022).

### Bile acids and amyotrophic lateral sclerosis

Research on ALS, BAs, and BA receptors has progressed, focusing on associations between BA metabolic abnormalities and neuroprotection in ALS. Clinical studies indicate that TUDCA is safe and potentially efficacious in patients with ALS (Lo Giudice et al., 2023; Reggiardo et al., 2023). Two independent trials report that TUDCA can slow functional decline and improve survival in ALS. Mechanisms may involve suppressing endoplasmic-reticulum stress and mitigating mitochondrial dysfunction (Lo Giudice et al., 2023; Reggiardo et al., 2023). Retrospective studies report that oral TUDCA extends median survival in ALS, with greater effects when initiated earlier (Elia et al., 2016; Paganoni et al., 2020; Zucchi et al., 2023). Cellular studies provide mechanistic insights. For instance, a stem-cell screening platform identified BAs that desensitize motor neurons to ER stress (Thams et al., 2019). *In vivo*, TUDCA preserved neuromuscular-junction innervation in SOD1-G93A mice, mitigating an early pathological event in ALS (Thams et al., 2019). A study of BA precursors suggested a direct role in ALS pathology (Mukhutdinova et al., 2018). 24S-OHC inhibits neurotransmitter release at the neuromuscular junction in SOD1-G93A mice, associated with the regulation of nitric oxide signaling and stabilization of membrane lipid rafts (Mukhutdinova et al., 2018). In ALS excitotoxicity, this inhibitory effect on synaptic transmission may represent an endogenous neuroprotective mechanism (Mukhutdinova et al., 2018). This suggests that, in addition to peripheral BA dysregulation, disturbances in brain cholesterol and BA metabolism may be intrinsic to ALS pathogenesis. In a cellular model of SOD1-related neurodegeneration, GUDCA reduced activation of matrix metalloproteinase-9 and caspase-9, mediators of neuroinflammation and apoptosis, respectively (Launay et al., 2017). Based on TUDCA studies, AMX0035 (Relyvrio) was developed (Paganoni et al., 2020). AMX0035 combines sodium phenylbutyrate and taurursodiol (pharmaceutical TUDCA). In clinical trials, AMX0035 modestly reduced functional decline in ALS (Paganoni et al., 2020). However, AMX0035 highlighted challenges in translating preclinical findings into clinical efficacy. A larger Phase 3 trial (PHOENIX) failed to confirm these benefits, leading to voluntary market withdrawal in 2024. Despite these results, the trajectory of AMX0035 emphasizes the rationale for targeting TUDCA-regulated pathways, such as ER stress and mitochondrial dysfunction, and points toward more refined treatment strategies for ALS.

In ALS, abnormal serum and fecal levels of specific BAs are observed. One study reported reduced serum concentrations of DCA and CDCA. Altered fecal BA profiles correlate with cognitive impairment, suggesting that BA dysmetabolism contributes to nervous system pathology (Gong et al., 2023). The gut microbiota appears to be a crucial component. In ALS, the gut microbiota shows diminished capacity for BA metabolism. This results in impaired conversion of primary to secondary BAs. This depletion is significant because secondary BAs are key ligands for intestinal receptors; thus, their absence likely disrupts gut homeostasis, particularly by impairing FXR signaling. As discussed earlier, FXR signaling is critical for maintaining barrier integrity and suppressing inflammation. Insufficient ligand-dependent activation could exacerbate gut-derived pathology and influence disease progression through MGBA pathways (Zhang et al., 2021c).

Compared with other major neurodegenerative diseases, direct links between BA signaling and ALS pathology are relatively understudied. However, the therapeutic trajectory of TUDCA illustrates both promise and challenges. The TUDCA-based co-formulation AMX0035 initially showed modest slowing of functional decline in the Phase 2 CENTAUR trial, with long-term data suggesting a survival benefit, leading to accelerated FDA approval (Paganoni et al., 2020, 2022). However, a larger Phase 3 confirmatory trial (PHOENIX) failed to replicate these findings, ultimately leading to the voluntary withdrawal of the drug from the market.

This outcome does not negate the rationale for targeting BA-modulated pathways. Instead, it highlights the need for in-depth studies to better understand BA signaling in ALS. Exploring therapeutic strategies, such as identifying responsive subgroups or developing combination therapies, remains a promising avenue for research.

## Microbiota–Gut–Brain Axis: A Central Hub for Bile Acid–Mediated Neuromodulation

### Dysregulation of the microbiota-gut-brain axis in neurodegenerative diseases

**Figure 5:**
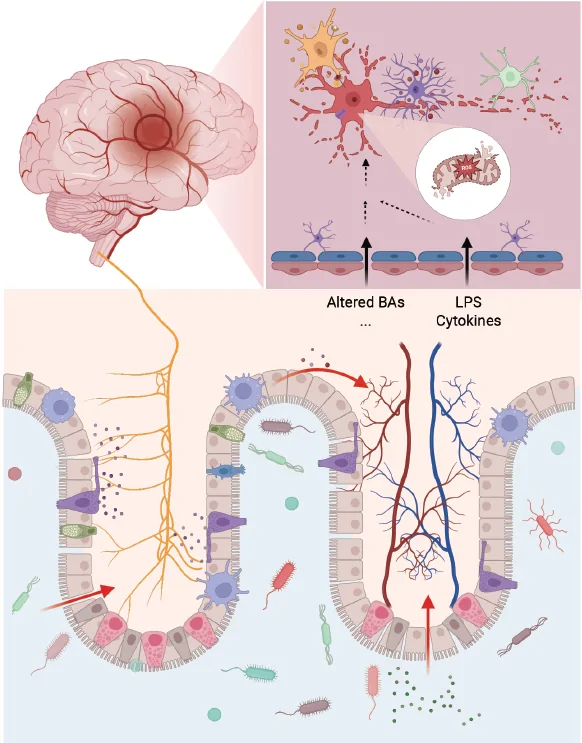
Key communication pathways of the microbiota–gut–brain axis. The microbiota–gut–brain axis is a bidirectional communication network linking the gut and the brain. Gut dysbiosis and increased intestinal permeability allow signaling molecules to affect the CNS via three main routes: (1) Humoral pathway—microbial metabolites such as BAs and pathogen-associated molecular patterns cross the intestinal barrier and BBB to directly influence brain cells; (2) Immune pathway—molecules such as LPS activate gut immune cells, leading to the release of pro-inflammatory cytokines that circulate to the brain and induce neuroinflammation; (3) Neural pathway—signals are transmitted from the gut to the brain via the vagus nerve, modulating neural activity and inflammation. These pathways converge to activate glial cells (microglia and astrocytes), trigger neuroinflammation, and promote oxidative stress and mitochondrial dysfunction, ultimately contributing to neuronal injury and the progression of neurodegenerative diseases. BAs: Bile acids; BBB: blood–brain barrier; CNS: central nervous system; LPS: lipopolysaccharide.

The MGBA is a bidirectional communication network comprising the gut microbiota, intestinal barrier, immune system, nervous system (including the vagus nerve and ENS), and microbial metabolites such as SCFAs, BAs, and neurotransmitters (**Figure 5**; Cryan et al., 2019). Core mechanisms include microbiota-derived metabolites regulating host immune responses, neurotransmitter synthesis, and barrier function. For instance, SCFAs maintain barrier integrity by modulating tight-junction proteins and influence CNS inflammation as histone deacetylase inhibitors (Dalile et al., 2019; Mou et al., 2022). Across neurodegenerative diseases, MGBA dysregulation shows common features, including: 1) reduced gut microbiota diversity (dysbiosis) in AD, PD, and ALS, with fewer beneficial bacteria and more pro-inflammatory taxa (Cryan et al., 2019; Mou et al., 2022); 2) increased intestinal permeability (“leaky gut”), which allows microbial components such as LPS to enter the bloodstream. This triggers peripheral immune activation, initiating a pro-inflammatory cytokine cascade that can compromise BBB permeability and accelerate the deposition of neurotoxic proteins such as Aβ and α-syn within the CNS (Dong, 2020; Fabi, 2024). Neuroinflammation is often driven by microbial metabolites. These metabolites can activate microglia through nuclear receptors such as FXR and receptors including toll-like receptor 4 (TLR4). This promotes sustained release of pro-inflammatory cytokines such as TNF-α and IL-1β, fostering a chronic inflammatory microenvironment in the CNS (Park and Gao, 2024). The BA system functions as a key signaling node within the MGBA, where it appears to exert dual regulatory functions in neurodegeneration. Through microbial 7α-dehydroxylation, primary BAs are converted into neuroactive secondary BAs. These metabolites modulate barrier integrity via FXR and can cross the BBB to activate brain TGR5. They can thereby regulate neuroinflammation and synaptic plasticity (Mulak, 2021; Ma et al., 2024;). In summary, this multilayered regulatory cascade along the gut microbiota-BA-brain axis reveals molecular mechanisms that underscore the pivotal role of the MGBA in the pathogenesis of neurodegenerative diseases.

### Bidirectional crosstalk between gut microbiota and bile acids

As core MGBA signaling molecules, BAs regulate systemic metabolic balance and nervous system function through a multi-receptor-mediated network. Their biological basis encompasses a pathway of synthesis, transformation, and signal transduction. Primary BAs (e.g., CA, CDCA) are synthesized in the liver and modified by gut microbiota (e.g., Clostridium, Bacteroides) through processes such as 7α-dehydroxylation into secondary BAs (e.g., LCA, DCA). This transformation increases BA chemical diversity and signaling activity (Wang et al., 2024a). BAs act in the intestine, liver, and CNS by binding nuclear receptors such as FXR, membrane receptors such as TGR5, and other receptors, including VDR, PXR, and CAR (Fiorucci et al., 2021).

In the CNS, BA actions across diseases involve three mechanisms: (1) Immune-inflammatory regulation: TGR5 activation in macrophages can inhibit the NLRP3 inflammasome, mitigating neuroinflammation. FXR can reduce the release of pro-inflammatory cytokines (e.g., IL-1β, TNF-α) by inhibiting TLR4 signaling (Fiorucci et al., 2024). Different BA subtypes can have opposing effects: UDCA inhibits microglial activation, whereas DCA may exacerbate neuroinflammation by activating TLR2/4 (Zhang et al., 2024). In the CNS, BA actions across diseases involve three mechanisms: (2) Neuroprotection versus toxicity: Certain BAs (e.g., TUDCA, GCDCA) can cross a compromised BBB and act on neurons and glia. TGR5 activation can promote astrocytic brain-derived neurotrophic factor secretion, whereas FXR mitigates oxidative stress by upregulating antioxidant genes (e.g., *SOD2*). Hydrophobic BAs, such as LCA, can increase mitochondrial membrane permeability and trigger neuronal apoptosis (Silva et al., 2022; Ni et al., 2025). (3) Microbiota-host metabolic interplay: BA dysmetabolism resulting from gut dysbiosis (e.g., imbalanced secondary BA ratios) can influence CNS neurotransmitter synthesis (e.g., 5-hydroxytryptamine [5-HT], γ-aminobutyric acid [GABA]) via the vagus nerve or systemic circulation. It can also promote the aggregation of pathological proteins such as α-syn and Aβ (Kim et al., 2021; Rob et al., 2025). These findings suggest that targeting BA receptors (e.g., FXR/TGR5 dual agonists) or modulating the gut microbiota-BA-brain axis may offer cross-disease therapeutic strategies.

Growing evidence indicates that gut microbiota-BA interaction is a regulatory hub within the MGBA. In this bidirectional pathway, the microbiota deconjugates and 7α-dehydroxylates liver-derived primary BAs, producing secondary BAs. Conversely, BAs modulate microbial homeostasis and metabolism via direct antimicrobial activity and activation of host receptors such as FXR and TGR5 (Ekwudo et al., 2025). This bidirectional crosstalk contributes to the pathogenesis of neurodegenerative diseases such as AD and PD. It influences neurological function through metabolic and signaling pathways.

In the “microbiota to BA” direction, microbial metabolism predominates. It shapes the composition and functional landscape of the BA pool. This occurs through several microbial transformations. A major transformation is deconjugation, which is primarily mediated by BSH enzymes expressed by genera such as *Bacteroides*, *Clostridium*, *Lactobacillus*, *Bifidobacterium*, and *Enterococcus* (Song et al., 2019). Oxidation and epimerization alter BA hydroxyl configuration and stereochemistry. These reactions are attributed to taxa including *Ruminococcus spp*., *Eggerthella lenta*, and *Clostridium spp.* (Ridlon and Gaskins, 2024). A key process is 7α-dehydroxylation. It generates secondary BAs and is largely carried out by Clostridium species such as *Clostridium scindens* via the Hylemon–Björkhem pathway (Ridlon et al., 2020). Other modifications include C3-dehydroxylation by Clostridium sporogenes and probable C12-dehydroxylation by *Bacteroides spp.* (Ridlon and Gaskins, 2024). These microbial activities are not static. They may become dysregulated in neurodegenerative diseases. Such shifts reshape the intestinal BA pool, altering composition, circulating bioavailability, and downstream signaling potential. A multicenter cohort in patients with AD underscores this point. Serum levels of the primary BAs, CA, were reduced. DCA and derivatives (secondary BAs) were elevated. An increased DCA: CA ratio correlated with greater cognitive impairment. This implicates enhanced 7α-dehydroxylation activity in the pathological gut microenvironment of AD (Mahmoudian Dehkordi et al., 2018). APP transgenic mouse models support this link, showing that Aβ accumulation can induce gut microbiota changes, such as increased *Klebsiella*, associated with abnormal BA metabolism (Chin et al., 2025). This suggests a vicious cycle in which AD pathology and gut microbiota-BA dysregulation reinforce each other, a pattern that may differ from other neurodegenerative conditions.

Similar patterns have emerged in PD. Analyses of appendiceal tissue revealed elevated DCA and LCA, toxic secondary BAs linked to *Clostridium*-mediated conversion pathways (Li et al., 2021). Clinical studies echo this disruption of secondary BA homeostasis. For example, cholecystectomy, which alters BA flow and gut microbiota, is associated with increased PD risk (Kim et al., 2021). Furthermore, patients with α-synucleinopathy, particularly PD, show a greater reduction in SCFA-producing bacteria such as *Ruminococcus*, alongside altered BA profiles. This points to specific gut microbiota–BA–brain axis impairment in PD that contributes to neuroinflammation (Nishiwaki et al., 2022). In PD mouse models, *Clostridium innocuum* was identified as a negative regulator of the neuroprotective BA GUDCA (Shang et al., 2025). Its BSH activity suppresses GUDCA availability; inhibiting this species restores GUDCA levels and yields neuroprotective effects (Shang et al., 2025). Collectively, these findings highlight the modulatory influence of gut microbial enzymes, particularly BSH and 7α-dehydroxylase, on BA profiles, suggesting a mechanistic link between microbiota-derived BA modulation and CNS outcomes.

The pattern observed in AD and PD—the accumulation of harmful secondary BAs—is not the only form of dysbiosis in the gut microbiota-BA-brain axis. The “disease-specific” nature of this axis is most strongly demonstrated in its association with MS. As a disease driven by autoimmunity and neuroinflammation, MS shows a gut microbiota-BA dysbiosis pattern that contrasts with, and may oppose, that in AD. In MS, a core issue is loss of protective signaling due to depletion of secondary BAs. Patients with MS exhibit microbiota dysbiosis, including reduced bacteria involved in BA metabolism (Antonini Cencicchio et al., 2025). This results in reduced levels of secondary BAs such as DCA and LCA (Wang et al., 2024a). This is relevant because these BAs can promote differentiation of immunosuppressive regulatory T cells, a process important for maintaining immune tolerance (Wang et al., 2024a; Antonini Cencicchio et al., 2025). Therefore, microbially driven depletion of these immunoregulatory BAs is thought to impair control of autoimmunity, exacerbating neuroinflammation in MS. By contrast, AD is characterized by an overabundance of certain BAs, leading to enhanced toxic signaling. Patients with AD often exhibit elevated secondary BAs, including DCA, and an elevated DCA: CA ratio is associated with cognitive decline (Mahmoudian Dehkordi et al., 2018). In animal studies, DCA also contributes to AD pathology (Li et al., 2024b). A direct comparison between MS and AD reveals two different patterns of gut microbiota-BA-brain axis dysfunction. This indicates the importance of defining disease-specific metabolic signatures to develop targeted diagnostic and therapeutic strategies.

Conversely, BAs govern gut-microbiota structure and function, illustrating the “BA to microbiota” axis. Owing to their lipophilicity, BAs exert direct antimicrobial effects. High concentrations, particularly of hydrophobic secondary BAs, typically inhibit Gram-negative bacteria (e.g., *Bacteroides*) while enriching Gram-positive populations (e.g., *Clostridium*) (Graham et al., 2018). When intestinal BA levels decrease, Gram-negative LPS-producing bacteria such as *Bacteroides* may become relatively more abundant (Mulak, 2021; Ehtezazi et al., 2023). Beyond direct antimicrobial actions, BAs act as signaling molecules. They activate FXR and TGR5 in intestinal epithelial and immune cells, guiding antimicrobial-peptide production, regulating inflammation, and maintaining barrier integrity. For example, secondary BAs promote autophagy via TGR5, whereas primary BAs bind FXR, suppress pro-inflammatory responses in macrophages, and strengthen the intestinal barrier (Kiriyama and Nochi, 2023b; Shatova et al., 2023). In ALS, gut dysbiosis may initiate disruption of BA homeostasis. Impaired FXR/TGR5 signaling could compromise immunomodulatory and neuroprotective mechanisms (Zhai et al., 2024). For instance, reduced butyrate-producing bacteria (e.g., *Ruminococcus*, *Blautia*) in ALS could weaken SCFA-mediated regulation of BA metabolism. The resulting disturbances in BA signaling can alter inflammatory states via the FXR/TGR5 pathway (Ayala et al., 2025). In summary, BAs shape gut-microbiota composition and function through direct antimicrobial effects and receptor-mediated signaling, achieving bidirectional regulation.

Taken together, gut microbiota–BA interaction is tightly interwoven. When this relationship is dysregulated in neurodegenerative diseases, it likely links gut pathology to CNS damage. This pattern is observed across several neurodegenerative diseases. In AD, BA profiles shift—with reduced primary and elevated secondary BAs—and correlate with cognitive decline (Mahmoudian Dehkordi et al., 2018). A similar pattern is seen in PD. Elevated levels of toxic secondary BAs are observed in the gut. Specific bacteria, such as *Clostridium innocuum*, can reduce the production of beneficial BAs such as GUDCA (Shang et al., 2025). In HD, alterations in gut microbiota composition and BA metabolism genes suggest that the BA pathway contributes to HD-related neuroinflammation (Ekwudo et al., 2025). These disease-specific alterations underscore the importance of the gut microbiota–BA–brain axis in pathology. A deeper understanding of these pathways is important. This may open avenues for developing early diagnostic biomarkers and targeted therapies. This axis is a pillar of the MGBA and influences the onset and progression of neurodegenerative diseases. Further exploration may identify targets for clinical interventions.

### Interactions of the bile acid system with other microbiota–gut–brain axis signaling pathways

The BA system does not operate in isolation within the MGBA but interacts with multiple other signaling pathways. BAs are closely related to SCFAs; many gut bacteria, such as *Clostridium* and *Bacteroides*, both promote BA metabolism and produce SCFAs (Yang et al., 2024a). This dual activity enables BAs and SCFAs to act synergistically to maintain barrier function, regulate inflammation, and provide neuroprotection (Kiriyama and Nochi, 2023a). For example, in intestinal epithelial cells, primary BAs via FXR and butyrate cooperatively enhance tight-junction protein expression. This helps maintain barrier integrity, reducing leakage of neurotoxic metabolites into the circulation (He and You, 2020).

The BA system is also linked with neurotransmitter metabolism. Studies report that BAs influence the production of 5-hydroxytryptamine, GABA, and dopamine precursors by affecting gut-microbiota composition (Wu et al., 2022; Darmanto et al., 2025; Xie et al., 2025). Of particular note is the interaction between BAs and tryptophan metabolism, especially the regulation of the kynurenine pathway (Lin et al., 2024). Tryptophan metabolites such as 5-HT and kynurenine can indirectly influence the enterohepatic circulation of BAs by modulating gut motility or inflammatory responses (Zhang et al., 2021b; Yu et al., 2024).

## Sex as a Biological Variable: Dimorphic Regulation of the Microbiota–Bile Acids–Brain Axis

### Sex-specific differences in bile acid profiles

The MGBA is a central network governing the interplay between intestinal health and neurodegenerative diseases. Although this axis is conserved, its activity and outcomes vary across populations, with sex being an often-underestimated biological variable that modulates its multiple nodes.

Sex differences in BAs reflect metabolic distinctions between males and females. Males typically display lower TBA concentrations than females (Fitzinger et al., 2024). This pattern also appears in disease states. Females with non-alcoholic fatty liver disease (NAFLD) have elevated TBA, primary BAs, and glycine-conjugated BAs compared with healthy counterparts (Trottier et al., 2011).

These sex-based variations extend to individual BA subtypes. Male plasma typically contains higher concentrations of the cholesterol precursor 26-hydroxycholesterol (26-OHC), along with CDCA and GUDCA. However, the GUDCA difference may attenuate after adjusting for body-fat percentage (Reinicke et al., 2018; Osuna-Prieto et al., 2021). Females consistently show elevated concentrations of certain hydrophobic, potentially cytotoxic BAs. For example, in metabolic dysfunction–associated steatotic liver disease (MASLD), females have higher levels of LCA and derivatives (e.g., GLCA, TLCA) than males (Lyu et al., 2025). Levels of taurine-conjugated BAs, such as TCA and TCDCA, also tend to be higher in females (Reinicke et al., 2018; Lyu et al., 2025).

These compositional differences are linked to sex-specific metabolic pathways. A key distinction is the primary-to-secondary BA ratio, typically higher in males than in females (Lyu et al., 2025). This likely reflects microbiotal conversion of primary BAs. Females with a functional microbiota show higher circulating secondary BA levels than males. This highlights a sex-specific, microbiota-mediated metabolic regulation (Ruan et al., 2022). Conjugated BA metabolism also shows sex-based variations. For example, in a type 2 diabetes mellitus (T2DM) model, conjugated BAs increase in both male and female mouse livers, but regulation appears to occur through distinct pathways (Deng et al., 2024). Clinical data suggest that glycine-conjugated BAs accumulate more readily in females (Lyu et al., 2025). Overall, variations in concentration, composition, and metabolic pathways define BA sexual dimorphism. This provides a biological basis for sex-specific responses to metabolic diseases and pharmacotherapy.

### Mechanisms of bile acid sexual dimorphism

This pronounced sexual dimorphism in BA metabolism stems from a multilevel network involving endocrine signaling, gut microbiota dynamics, and host metabolic pathways, with sex hormones acting as the primary regulators. Androgens, especially testosterone, exert dual regulatory effects. They can indirectly modulate the gut microbiota composition through BA signaling pathways. They also directly regulate host metabolic gene expression. Together, these actions shape sex-specific BA profiles (Gathercole et al., 2022; Duan et al., 2024). In the liver, androgens directly regulate BA synthesis. They alter the activity of enzymes such as aldo-keto reductase 1D1 (AKR1D1) and CYP7A1. As a result, BA homeostasis and related metabolic pathways differ between males and females (Gathercole et al., 2022).

The gut microbiota acts as an orchestrator of this sexual dimorphism, especially in secondary BA differentiation. Female gut microbiota are more efficient at converting primary to secondary BAs. This results in higher circulating secondary BAs in females than in males, and the difference disappears in germ-free mice. This supports a central regulatory role for the microbiota (Baars et al., 2018; Murali et al., 2022). Furthermore, host–microbiota interactions modulate BA conjugation in a sex-dependent manner (Reinicke et al., 2018; Ma et al., 2020). This divergence likely stems from sex-specific expression patterns of microbial BA hydrolases.

Host hepatic metabolic pathways also differ by sex. This is evident under pathological conditions such as NAFLD. In these cases, female livers tend to synthesize primary BAs, whereas males tend to accumulate secondary BAs. This may reflect sex-specific expression of rate-limiting enzymes, including CYP7A1 and CYP8B1 (Fitzinger et al., 2024; Lyu et al., 2025). Hepatic BA-transporter activity also shows sex-based variations. For example, chenodeoxycholic acid inhibited OATP4C1 more strongly in males, which could affect BA reabsorption and sex-dependent drug metabolism (Yamauchi et al., 2022).

Taken together, these mechanisms shape a BA pool intertwined with the broader lipid metabolism network. A split emerges: male BA pools predominantly feature 12α-hydroxylated BAs such as CA, whereas female BA pools center on non-12α-hydroxylated BAs such as CDCA. This split drives differences in physicochemical properties and signaling (Zhang et al., 2021a; Lyu et al., 2025). In AD models, this distinction propagates through downstream processes, mainly via FXR and TGR5 signaling (Kaur et al., 2021; Chen et al., 2023c; Golonka et al., 2024). Ultimately, interacting mechanisms—hormonal regulation, microbial composition, and metabolic pathways—drive sex-specific differences. These factors help explain divergent responses in aging, disease progression, and treatment outcomes.

### Clinical implications of sex-specific bile acid metabolism

These sex-based differences in BA metabolism have significant clinical relevance, as they can drive variations in disease risk, treatment responses, and long-term outcomes. This metabolic sexual dimorphism offers a crucial perspective for understanding sex bias in metabolic, oncological, and reproductive diseases. They may also provide sex-specific biomarkers for risk assessment. For metabolic diseases such as NAFLD/ metabolic dysfunction–associated steatotic liver disease, female patients typically have higher levels of TBAs, primary BAs, and glycine-conjugated BAs. Secondary BA levels (e.g., LCA, GLCA, TLCA) also exceed those in males. This is consistent with the female predisposition toward cholestatic pathology (Fitzinger et al., 2024; Lyu et al., 2025). In contrast, male patients tend to show a higher primary to secondary BA ratio. This shift may increase susceptibility to secondary BA-driven inflammation. Similar sex-specific patterns of BA accumulation are observed in T2DM models (Ruan et al., 2022). These findings suggest that elevated secondary BAs in females could serve as a risk marker for fatty-liver progression. In males, postoperative levels of 12α-hydroxylated BAs might indicate metabolic improvement.

In cancer and reproductive disorders, BA sex differences are also relevant. BA profiles are hypothesized to contribute to the higher incidence of hepatocellular carcinoma in males; hormone-like effects may contribute to the sex bias (Golonka et al., 2024). In polycystic ovary syndrome, conjugated primary BAs are elevated in follicular fluid and serum and correlate positively with androgen levels. This suggests that BAs may be involved in hyperandrogenic pathology and could serve as surrogate markers of androgen activity (Zhang et al., 2019; Yang et al., 2021). BAs also regulate human germ cells and may be linked to unexplained infertility (Buchinger et al., 2019).

The sex-specific nature of BA metabolism warrants sex-stratified reassessment and redesign of drug therapies, a cornerstone of precision medicine. Therapeutic responses exhibit sex differences. For example, in cholestatic liver diseases, fenofibrate shows hepatoprotective effects in males (18.6% reduction in TBAs), whereas these benefits are largely absent in females (Trottier et al., 2011). This implies that efficacy, dosing, and monitoring parameters may require sex-stratified protocols for certain therapies. Similarly, bariatric procedures influence BA metabolism in a sex-dependent manner. For instance, Roux-en-Y gastric bypass increases 12α-hydroxylated BAs in males, whereas sleeve gastrectomy does not show a notable sex-specific effect (Zhang et al., 2021a).

Accumulating evidence has implications for drug development. Therapies targeting BA signaling, such as FXR and TGR5 agonists, are unlikely to produce uniform effects across populations. Among variables driving this divergence, sex is a major determinant. Therefore, clinical trials should incorporate sex-stratified analyses from the outset. Without this, the understanding of efficacy and safety remains incomplete. These differences are dynamic rather than fixed. They shift across the lifespan, particularly in mid to older age. With aging, declining sex hormones reshape the gut environment and alter hepatic enzyme expression. Aging may compound pre-existing metabolic tendencies, leading to divergent physiological trajectories in males and females.

A better understanding of these dynamic changes could enable tailored interventions. For example, nutritional strategies or preventive medications could be designed to address the specific metabolic profiles of a 65-year-old woman versus a man of the same age. Achieving this level of precision would advance personalized medicine from a slogan to a clinical reality.

### Sex-specific bile acid signaling in neurodegenerative diseases

Growing evidence suggests that sex-specific BA metabolism may influence susceptibility to neurodegenerative diseases through systemic and central mechanisms. Differences in serum BA levels between sexes may alter systemic inflammation and BBB integrity, factors that play roles in pathogenesis. Sex-specific differences in serum BA profiles are reported in AD, and postmenopausal estrogen declines may exacerbate these differences (Hurley et al., 2022; Chen et al., 2023c). This may contribute to higher AD prevalence in females through BA dysregulation and enhanced neuroinflammation (Hurley et al., 2022; Chen et al., 2023c). Specifically, elevated hydrophobic BAs such as DCA upregulate TGR5 in AD and may potentiate BBB disruption and pro-inflammatory pathways (Li et al., 2024b).

In AD, BA dysregulation is heterogeneous; this appears especially true in male patients (Chen et al., 2023c). BA profiles often shift with metabolic inflammation and heightened oxidative stress—changes some researchers suggest may underlie a neuroinflammatory pattern specific to females (Chen et al., 2023c). Animal studies offer additional support. In several AD models, altered BA levels amplify oxidative stress and activate inflammatory cascades in the brain, potentially explaining sex-dependent differences in cognitive trajectories (Kaur et al., 2021; Lirong et al., 2022).

Sex hormones are the primary upstream drivers of this BA sexual dimorphism. They act through two routes: directly regulating hepatic BA-synthetic enzymes and remodeling the gut microbiota, the principal source of secondary BAs (Gathercole et al., 2022; Hurley et al., 2022; Li et al., 2022; Duan et al., 2024; Reichardt et al., 2025). This peripheral BA sexual dimorphism can influence brain function, and a key potential mechanism is direct molecular crosstalk between BA and sex hormone signaling pathways. Evidence from peripheral tissues shows that BA signaling (e.g., FXR) intersects with sex-hormone pathways (e.g., ERα), and some hydrophobic BAs can bind estrogen receptors directly (Ehtezazi et al., 2023; Golonka et al., 2024). Because BA and sex-hormone receptors are co-expressed in neurons and glia, this crosstalk may also operate within the CNS.

We propose a testable hypothesis: by orchestrating the formation of a sex-specific peripheral BA pool through dual regulation of hepatic synthesis and gut microbiota metabolism, sex hormones create a unique BA signaling signature. This signature, upon entering the CNS, acts on local BA receptors to modulate key pathological processes such as neuroinflammation and oxidative stress, ultimately contributing to the sex-specific susceptibility and progression of neurodegenerative diseases. Direct evidence for this pathway is currently lacking, but its causality can be tested using sex-difference models (e.g., ovariectomy, androgen-receptor knockout).

## Limitations

Translational and methodological limitations further complicate progress. Preclinical research is hampered by significant interspecies differences in BA composition and receptor distribution between animal models and humans, limiting the predictive value of previous studies (Verkade et al., 2023; Gillard and Leclercq, 2024). Methodologically, current analytical techniques such as conventional LC-MS often lack the sensitivity to comprehensively map the low-abundance intracerebral BA metabolome, obscuring the full picture of local BA signaling in the brain (Shansky and Bespyatykh, 2022; Li et al., 2025b). Moreover, a reliance on cross-sectional studies and end-stage specimens creates a longitudinal data deficit, making it difficult to determine whether BA alterations are a cause or a consequence of disease.

Finally, the critical role of sex as a biological variable represents both a challenge and an opportunity. As drugs such as fenofibrate demonstrate, therapeutic efficacy and toxicity profiles can be sex-specific. Omitting sex-stratified analyses in the development of BA modulators constitutes a critical yet avoidable hazard, potentially masking benefits in one sex or exposing the other to unforeseen risks.

## Dual Role of the Bile Acid System: Therapeutic Potential, Challenges, and Prospects

### Clinical translation: Current status and lessons learned

In recent years, drugs targeting the BA system have shown promise in metabolic and immune diseases. For example, the FXR agonist OCA is FDA-approved for primary biliary cholangitis and has entered Phase III trials for nonalcoholic steatohepatitis (NCT02548351). FXR, a core target of BA signaling, has attracted extensive clinical interest. Several FXR agonists are in clinical studies, including EDP-305 for nonalcoholic steatohepatitis; a 12-week Phase II study showed that EDP-305 reduced ALT and liver fat (NCT03421431). CS0159, a nonsteroidal FXR agonist from Cascade, demonstrated efficacy and a favorable safety profile in a Phase II PBC study (NCT05591079). Norucholic acid, an engineered BA derivative, is being evaluated in primary sclerosing cholangitis: in a Phase III trial (NUC-5) in 86 patients, interim week-96 results (planned 192 weeks) showed statistically significant improvements versus placebo in primary and key secondary endpoints, with more patients achieving treatment goals and histological improvement in liver fibrosis without progression (NCT03872921).

In stark contrast to advances in metabolic diseases, the exploration of BA-targeted therapies in neurodegenerative diseases remains in its early stages. A prime example is AMX0035: it initially showed signals of slowed progression in ALS (NCT03127514) and received conditional FDA approval, but a subsequent Phase III trial (NCT05021536) failed to confirm efficacy, leading to its market withdrawal. This underscores that early signals require confirmation in adequately powered, rigorously designed Phase III trials before clinical application. Beyond AMX0035, several BAs or derivatives are under evaluation. In MS, a Phase II study (NCT03423121) found TUDCA to be well-tolerated and suggested effects on inflammation and neuroprotection, warranting larger trials with clinical endpoints. In PD, the UK UDCA-PD study (NCT03840005) showed that high-dose UDCA improved biomarkers of mitochondrial energy metabolism and was generally well-tolerated. Although traditional motor scores did not improve significantly, the mitochondrial signal supports progression to longer, adequately powered studies. In AD, clinical exploration remains early, and no BA-related drugs have entered registrational trials.

Overall, compared with progress in peripheral systemic diseases, substantial translational gaps remain in neurodegeneration. Leveraging neuroprotective BA signaling is a key opportunity for future research. Subsequent sections address the challenges and potential paths forward.

### Therapeutic rationale: From biomarkers to disease-specific interventions

The clinical exploration of BA-based therapies is grounded in their potential as both diagnostic biomarkers and therapeutic agents. This dual utility stems from the distinct ways in which BA profiles are altered in specific neurodegenerative diseases, allowing for tailored intervention strategies.

#### Bile acids as biomarkers for early diagnosis and prognosis

A critical first step in clinical translation is identifying reliable biomarkers. Alterations in BA profiles, which often precede overt clinical symptoms, present a promising avenue for earlier disease identification and risk assessment. For instance, in a prodromal PD mouse model, reduced serum levels of protective BAs (TUDCA and UDCA) enabled a diagnostic model to distinguish diseased from control mice with high accuracy (AUC = 0.906), highlighting the potential for non-invasive early diagnosis (Graham et al., 2018). This potential is further supported by large-scale human cohort studies. An analysis of 1180 participants from the Alzheimer’s Disease Neuroimaging Initiative (ADNI) cohort demonstrated that BA profiles could enhance the accuracy of AD prediction (increasing AUC from 0.78 to 0.91) (Shao et al., 2020; Chen et al., 2023c). Crucially, this study also underscored the importance of sexual dimorphism: BA alterations were particularly prominent in males prior to diagnosis. This finding indicates that a single biomarker approach is likely insufficient; diagnostic models must integrate sex-specific biomarker combinations to achieve optimal accuracy and aid in personalized prognostic assessment (Chen et al., 2023c).

#### Disease-specific therapeutic strategies

Beyond their diagnostic value, the specific patterns of BA dysregulation in each disease provide a clear rationale for targeted therapeutic interventions (**Additional Table 4**). In AD, the typical profile of lower primary BAs and elevated neurotoxic secondary BAs provides a rationale for interventions aimed at reducing this toxic burden or restoring neuroprotective signaling (Mahmoudian Dehkordi et al., 2018). Preclinical studies show that BAs such as TUDCA and CDCA can favorably influence Aβ deposition, neuroinflammation, and insulin signaling (Lo et al., 2013; Bazzari et al., 2019; Wu et al., 2019a; Zangerolamo et al., 2021). The involvement of receptors such as TGR5 in Aβ generation further supports the development of targeted receptor modulators (Chen et al., 2019; Czarnecka et al., 2020; Shan et al., 2020; Li et al., 2024b). In PD, where patients also exhibit significant BA dysmetabolism, the therapeutic rationale centers on protecting dopaminergic neurons and enhancing mitochondrial function (Yakhine-Diop et al., 2020; Zhang et al., 2022). This is supported by studies showing that UDCA/TUDCA can improve mitochondrial function and reduce neuronal apoptosis, with early clinical studies showing signals of mitochondrial restoration (Payne et al., 2020; Luxenburger et al., 2022; Razavi et al., 2025). Other BAs such as CDCA may offer complementary anti-inflammatory benefits (Kaur et al., 2024). In ALS, TUDCA appears to slow disease progression and may extend survival. Mechanisms may include reduced endoplasmic-reticulum stress and improved mitochondrial function (Lo Giudice et al., 2023; Reggiardo et al., 2023). In HD, while research is preliminary, observed BA dysmetabolism provides a basis for exploring the neuroprotective potential of UDCA, TUDCA, and GUDCA (Huang et al., 2022; Chiang et al., 2024).

**Additional Table 4:** Preclinical and clinical evidence for BA-based therapies in neurodegenerative diseases

Drug	Disease	Type	Model	Dose & administration	Main findings	Reference
CDCA	AD	Animal	AD model rats	CDCA 90 mg/kg/d	CDCA lowered hippocampal BACE1 and Aβ_42_	Bazzari et al., 2019
TUDCA	AD	Animal	APP/PS1 mice	0.4% in diet, 6 mon	Improved spatial, recognition and contextual memory; reduced hippocampal and prefrontal amyloid plaques.	Lo et al., 2013
	PD	Animal	MPTP-induced mice	50 mg/kg, i.p., for 3 d	Protected nigral dopaminergic neurons, reduced fiber loss, improved motor functions.	Castro-Caldas et al., 2012
	PD	Animal	MPTP-induced mice	500 mg/kg, i.p., for 35 d	Prevented dopaminergic neuron loss in SNpc, preserved striatal dopamine, improved motor function, and reduced neuroinflammation.	Cuevas et al., 2022
	ALS	Animal	SOD1-G93A mice	500 mg/kg, i.p., every 3 d × 7	Enhanced neuromuscular innervation, slowed motor neuron degeneration.	Thams et al., 2019
	ALS	Clinical	ALS patients	2 g/d orally, 1 yr	Adjunct to riluzole slowed ALSFRS-R decline (~4-5 mon survival benefit).	Elia et al., 2016
	HD	Animal	R6/2 mice	500 mg/kg, s.c., 6 wk	Reduced striatal apoptosis and atrophy, inhibited huntingtin inclusions, improved motor/sensorimotor deficits.	Keene et al., 2002
	MS	Animal	MOG35-55 EAE mice	500 mg/kg orally, from onset to 28 d	Reduced clinical score, demyelination, microglia/macrophage infiltration, astrocyte reactivity.	Bhargava et al., 2020
	MS	Clinical	progressive MS patients	2 g/d orally, 16 wk	Central memory CD4 and Th1/17 cells decreased, CD4 naive cells increased	Ladakis et al., 2025
UDCA	PD	Animal	MPTP-induced mice	400 mg/kg/d orally, 4 wk	Preserved dopaminergic neurons, improved motor function, increased DA/5-HT, antioxidant enzymes, and reduced proinflammatory cytokines.	Jiang et al., 2022
	PD	Clinical	Early PD patients	30 mg/kg/d, orally, 48 wk	Well tolerated; ^31^P-MRS showed increased Gibbs free energy and Pi in basal ganglia, suggesting improved neuronal bioenergetics.	Payne et al., 2023
	ALS	Clinical	ALS patients	1 g, twice daily, orally, 54 wk	Slowed ALSFRS-R decline compared with placebo.	Elia et al., 2016
6-ECDCA	MS	Animal	MOG35-55 EAE mice	10 mg/kg/d orally, 11 d	Reduced T/B cell activation, proinflammatory cytokines, VLA-4 expression; ameliorated disease.	Ho and Steinman, 2016

^31^P-MRS: Phosphorus-31 magnetic resonance spectroscopy; 5-HT: 5-hydroxytryptamine (Serotonin); 6-ECDCA: 6-ethylchenodeoxycholic acid; Aβ_42_: amyloid-β42; AD: Alzheimer's disease; ALS: amyotrophic lateral sclerosis; ALSFRS-R: ALS Functional Rating Scale-revised; APP/PS1: amyloid precursor protein/presenilin 1; BA: bile acid; BACE1: β-site amyloid precursor protein cleaving enzyme 1; CD4: cluster of differentiation 4; CDCA: chenodeoxycholic acid; DA: dopamine; EAE: experimental autoimmune encephalomyelitis; g: gram; HD: Huntington's disease; i.p.: intraperitoneal; kg: kilogram; mg: milligram; MOG: myelin oligodendrocyte glycoprotein; MPTP: 1-methyl-4-phenyl-1,2,3,6-tetrahydropyridine; MS: multiple sclerosis; PD: Parkinson's disease; R6/2: A transgenic mouse model of Huntington's disease; s.c.: subcutaneous; SNpc: substantia nigra pars compacta; SOD1-G93A: superoxide dismutase 1, G93A mutation; T/B cell: T lymphocyte/B lymphocyte; Th1/Th17: T helper 1/T helper 17; TUDCA: tauroursodeoxycholic acid; UDCA: ursodeoxycholic acid; VLA-4: very late antigen-4.

#### Principle of precision: Tailoring therapy to pathophysiology and time

Ultimately, these disease-specific profiles underscore a core principle: BA-based therapies cannot be “one-size-fits-all.” The therapeutic strategy must be tailored to the specific pathophysiology. This is best illustrated by the stark contrast between AD, which is associated with an accumulation of toxic BAs, and MS, which features a depletion of immunomodulatory secondary BAs (Mahmoudian Dehkordi et al., 2018; Li et al., 2024b; Wang et al., 2024a). A rational strategy for AD might involve reducing the toxic BA load, whereas in MS, replenishing specific BAs could be beneficial. Furthermore, the temporal dynamics of BA changes are critical. Alterations, such as the elevation of DCA in AD, can be early events in the disease cascade, suggesting that the timing of intervention is paramount (Li et al., 2024b). Ignoring these disease-specific and temporal complexities risks misdirecting therapeutic efforts. Acknowledging this complexity is the foundation for designing the next generation of truly precise and personalized BA-based interventions.

### Challenges and risks of bile acid–targeted therapy

Despite their promise, the application of BA-based therapies for the treatment of neurodegenerative diseases faces significant hurdles. These include the metabolic complexity of the BAs themselves, as well as methodological and translational challenges.

A major biological challenge is the intrinsic dual role of BAs. Many BAs, particularly hydrophobic ones, have a narrow therapeutic window where beneficial signaling can rapidly transform into cytotoxicity. This biphasic and context-dependent nature, as illustrated in **Additional Table 5**, means that therapeutic interventions can have unpredictable outcomes. Beyond this inherent complexity, systemic regulation also carries the risk of off-target effects, including hepatobiliary toxicity, disruption of metabolic homeostasis, and alterations in the gut microbiota (Awoniyi et al., 2023; Henry et al., 2023; Zhao et al., 2023a, b). In CNS, the BBB remains a formidable obstacle, limiting the penetration of most BAs (Macpherson et al., 2023; Jiao et al., 2025).

**Additional Table 5:** Biphasic and context-dependent characteristics of key BA

BA	Beneficial effect	Toxic effect	Mechanism/pathway	Disease	Reference
DCA	Enhances lipolysis and thermogenesis, improving obesity and insulin.	Induces low-grade intestinal inflammation, disrupts the barrier, activates NLRP3, promotes colorectal cancer, and exacerbates Aβ pathology via TGR5.	TGR5 agonist (promotes BAT thermogenesis), modest FXR agonist; upregulates ATGL, HSL, and UCP1 (lipolysis); activates NLRP3; promotes APP processing via TGR5.	Obesity, T2DM; CRC, IBD, AD	Liu et al., 2018; Li et al., 2024b; Tuerhongjiang et al., 2025
LCA	Protects the intestinal barrier; anti-inflammatory via VDR, SIRT1, and Nrf2; mimics calorie-restriction benefits (anti-aging) via AMPK activation.	Most hydrophobic and hepatotoxic BA (causes cholestasis); Nrf2 is required to mitigate LCA-induced liver injury.	Binds VDR to maintain tight junctions and inhibits NF-kB; activates SIRT1/Nrf2 and AMPK longevity pathways.	IBD, longevity; CLD	Tan et al., 2010; Venglovecz, 2016; Qu et al., 2025
CDCA	Most potent natural FXR agonist; improves lipid metabolism and insulin sensitivity; upregulates BSEP/OST to increase BA excretion.	Excess CDCA (cholestasis) activates NLRP3 via ROS and K^+^ efflux, driving IL-1β and liver fibrosis; induces IL-1β release from dendritic cells (pro-inflammatory)	Activates FXR/FGF19 axis to regulate BA homeostasis (suppresses synthesis and promotes efflux); triggers TGR5/ EGFR→ROS production and ATP release, activating NLRP3.	GS, MetS; CHB, LF	Gong et al., 2016; Guo et al., 2018; Oleszycka et al., 2023
UDCA	Choleretic (increases bile flow); reduces biliary cholesterol saturation; protects hepatocytes and cholangiocytes from hydrophobic BA toxicity by inhibiting ROS, acting as an antioxidant, and being anti- apoptotic.	Generally well tolerated; however, high-dose UDCA in PSC increased the risk of adverse outcomes.	Partial FXR agonist with immunomodulatory effects; upregulates bile acid (BA) efflux transporters; displaces toxic bile acids from the pool; activates antioxidant and anti-apoptotic pathways.	PBC, NAFLD; PSC	Imam et al., 2011; Roma et al., 2011

Aβ: Amyloid-β; AD: Alzheimer's disease; AMPK: AMP-activated protein kinase; APP: amyloid precursor protein; ATGL: adipose triglyceride lipase; ATP: adenosine triphosphate; BA: bile acid; BAT: brown adipose tissue; BSEP: bile salt export pump; CDCA: chenodeoxycholic acid; CHB: chronic hepatitis B; CLD: chronic liver disease; CRC: colorectal cancer; DCA: deoxycholic acid; EGFR: epidermal growth factor receptor; FGF19: fibroblast growth factor 19; FXR: farnesoid X receptor; GS: gallstones; HSL: hormone-sensitive lipase; IBD: inflammatory bowel disease; IL-1β: interleukin-1 β; K^+^: potassium ion; LCA: lithocholic acid; LF: liver fibrosis; MetS: metabolic syndrome; NAFLD: non-alcoholic fatty liver disease; NF- kB: nuclear factor-K B; NLRP3: NLR family pyrin domain containing 3; Nrf2: nuclear factor erythroid 2-related factor 2; OST: organic solute transporter; PBC: primary biliary cholangitis; PSC: primary sclerosing cholangitis; ROS: reactive oxygen species; SIRT1: Sirtuin 1; T2DM: type 2 diabetes mellitus; TGR5: G protein-coupled bile acid receptor 5; UCP1: uncoupling protein 1; UDCA: ursodeoxycholic acid; VDR: vitamin D receptor.

### Future research directions and a clinical translation roadmap

The BA system, as a metabolic link across neurodegenerative diseases, holds potential for research and clinical translation. First, a comprehensive analysis of the intracerebral BA metabolome is a key direction. This requires high-sensitivity multi-omics to map BA profiles across brain regions and monitor dynamics throughout disease progression. This will support the identification of disease-specific BA biomarkers, enabling early diagnosis and risk prediction. Second, a deep exploration of gut microbiota-BA-brain mechanisms is crucial. Specifically, it is essential to understand how microbially produced secondary BAs, such as DCA and LCA, modulate neuroinflammation and BBB function and how this pathway can be targeted therapeutically. Third, developing highly selective drugs targeting BA-receptor subtypes, such as brain-permeable FXR modulators or TGR5 agonists with reduced peripheral side effects, is a priority. Furthermore, overcoming the BBB barrier to delivery is critical to ensure efficacy and mitigate systemic side effects. A major risk of systemic BA intervention is disruption of the native enterohepatic circulation. Chronically elevating the systemic BA pool could interfere with feedback regulation of BA synthesis and gut-microbial composition, leading to unforeseen metabolic consequences. Future research should focus on innovative, brain-targeted delivery strategies. One approach is receptor-mediated transcytosis, using ligands for receptors such as the transferrin receptor and low-density lipoprotein receptor 1 to shuttle BA-loaded carriers into the brain (Terstappen et al., 2021; Mokarram et al., 2025; Porro et al., 2025). Additionally, nanotechnology-driven systems offer a platform: encapsulating therapeutic BAs such as TUDCA in carriers (liposomes, polymeric nanoparticles, exosomes) could enhance CNS penetration and bioavailability while minimizing systemic toxicity and off-target effects (Tian et al., 2024; Basyoni et al., 2025; Nikfar et al., 2025). Mitigating potential BA toxicity also warrants consideration (Moonen et al., 2025). These delivery technologies may enable precise and effective BA treatment in the brain.

For clinical translation, near-term efforts should prioritize repurposing approved BA drugs (e.g., UDCA, TUDCA) for neurodegenerative indications through accelerated trials. Mid-term strategies should focus on novel BA analogs and receptor modulators, such as 6-ethyl-CDCA (OCA) derivatives, to enhance neuroprotection while minimizing toxicity. One approach is combination therapy to circumvent the hepatotoxicity of monotherapy. This strategy has been explored in cholestatic liver diseases. The principle is to address mechanisms of BA toxicity through multi-target intervention (Yan et al., 2021; Klindt et al., 2024). Although risks (e.g., drug-drug interactions) must be considered, applying this “multi-target, multi-pathway” approach to neurodegeneration may yield safer, more effective treatments.

The long-term strategy is to develop precision BA therapy, tailoring interventions to BA metabolic profiles and genetic backgrounds. Clinical trials should be phased: first, test BA prevention in high-risk groups; next, assess progression in early patients; finally, evaluate symptom control in later stages. Furthermore, establishing international BA-metabolism databases and biobanks and integrating multicenter data will accelerate research and translation. To support translation, it is crucial to investigate causal relationships underlying sex differences in the BA system. Therefore, preclinical studies using sex-specific models (e.g., ovariectomy, androgen-receptor knockout) and, where appropriate, clinical studies are needed to investigate links between BA sexual dimorphism and disease susceptibility.

The BA system, as a metabolic link across neurodegenerative diseases, offers perspectives on pathogenesis and provides a basis for broad-spectrum neuroprotective strategies. With systematic research and strategic translation, BA-targeted therapy may become a paradigm in neurodegeneration treatment, enabling movement from symptom control to disease modification. A fundamental component of future research is the systematic study of sex as a biological variable. A systematic study of sex as a fundamental biological variable is therefore not just an option, but a prerequisite for progress. The future of precision BA-based therapy for neurodegenerative diseases will undoubtedly depend on a deep understanding of these sex differences.

## Additional files:

***Additional Table 1:*** *Key downstream signaling pathways of TGR5 in the CNS.*

***Additional Table 2:*** *Characteristics of additional BA receptors.*

***Additional Table 3:*** *Association between BAs and AD biomarkers.*

***Additional Table 4:*** *Preclinical and clinical evidence for BA-based therapies in neurodegenerative diseases.*

***Additional Table 5:*** *Biphasic and context-dependent characteristics of key BA.*

## Data Availability

*All relevant data are within the paper and its Additional files.*
